# QAFI: a novel method for quantitative estimation of missense variant impact using protein-specific predictors and ensemble learning

**DOI:** 10.1007/s00439-024-02692-z

**Published:** 2024-07-24

**Authors:** Selen Ozkan, Natàlia Padilla, Xavier de la Cruz

**Affiliations:** 1 Research Unit in Clinical and Translational Bioinformatics, Vall d’Hebron Institute of Research (VHIR), Universitat Autònoma de Barcelona, Barcelona, Spain; 2 Institució Catalana de Recerca i Estudis Avançats (ICREA), Barcelona, Spain

## Abstract

Next-generation sequencing (NGS) has revolutionized genetic diagnostics, yet its application in precision medicine remains incomplete, despite significant advances in computational tools for variant annotation. Many variants remain unannotated, and existing tools often fail to accurately predict the range of impacts that variants have on protein function. This limitation restricts their utility in relevant applications such as predicting disease severity and onset age. In response to these challenges, a new generation of computational models is emerging, aimed at producing quantitative predictions of genetic variant impacts. However, the field is still in its early stages, and several issues need to be addressed, including improved performance and better interpretability. This study introduces QAFI, a novel methodology that integrates protein-specific regression models within an ensemble learning framework, utilizing conservation-based and structure-related features derived from AlphaFold models. Our findings indicate that QAFI significantly enhances the accuracy of quantitative predictions across various proteins. The approach has been rigorously validated through its application in the CAGI6 contest, focusing on ARSA protein variants, and further tested on a comprehensive set of clinically labeled variants, demonstrating its generalizability and robust predictive power. The straightforward nature of our models may also contribute to better interpretability of the results.

**Supplementary Information:**

The online version contains supplementary material available at 10.1007/s00439-024-02692-z.

## Introduction

The transformative potential of Next-generation sequencing (NGS) for patients with genetic diseases is broadly recognized, yet its full impact is contingent upon two pivotal factors: the extent of its application and the accuracy of variant interpretation. Broadening the application of NGS could significantly enhance its lifesaving potential (Owen et al. 2023; Kingsmore et al. 2024), as illustrated in a study by Owen et al. (2023) that revealed that in a cohort of 112 infant deaths, a number of fatalities could have been prevented if rapid, diagnostic genome sequencing had been applied at the onset of symptoms or immediately upon ICU admission.

However, extending the frequency of NGS applications is not the sole challenge. A predominant issue lies in the complexity of interpreting the primary outcome of NGS experiments (Lázaro et al. 2021): the extensive list of genetic variants identified in a patient. Specifically, the process of determining the pathogenic or benign nature of each variant represents a formidable challenge, resulting, apart from classification errors, in a growing number of variants for which we cannot establish their impact (Tabet et al. 2022). These Variants of Uncertain Significance (VUS) limit the clinical yield of NGS and pose a significant barrier to its adoption as a standard diagnostic tool.

This situation has favored the advent of computational methods, generally based on machine learning algorithms, for pathogenicity prediction (Özkan et al. 2021). Initially met with caution, these models have since been integrated into clinical variant interpretation guidelines (Richards et al. 2015). They treat pathogenicity prediction as a classification problem in which a variant can be either pathogenic or benign. Specifically for missense variants—the focus of this paper—these tools may integrate a diverse range of properties into a single model (Jain et al. 2024a). This includes amino acid indexes such as hydrophobicity and volume changes, conservation-based metrics, like Shannon’s Entropy, as well as structure or biophysically related attributes, like accessibility and free energy estimates. The resulting predictive models are then trained, and their performance gauged, using sets of known pathogenic and benign variants. In recent years, the performances of these pathogenicity predictors have gradually increased (Jain et al. 2024a), leading to an important upgrade in the relevance given to their results in medical applications (Pejaver et al. 2022).

**Fig. 1:**
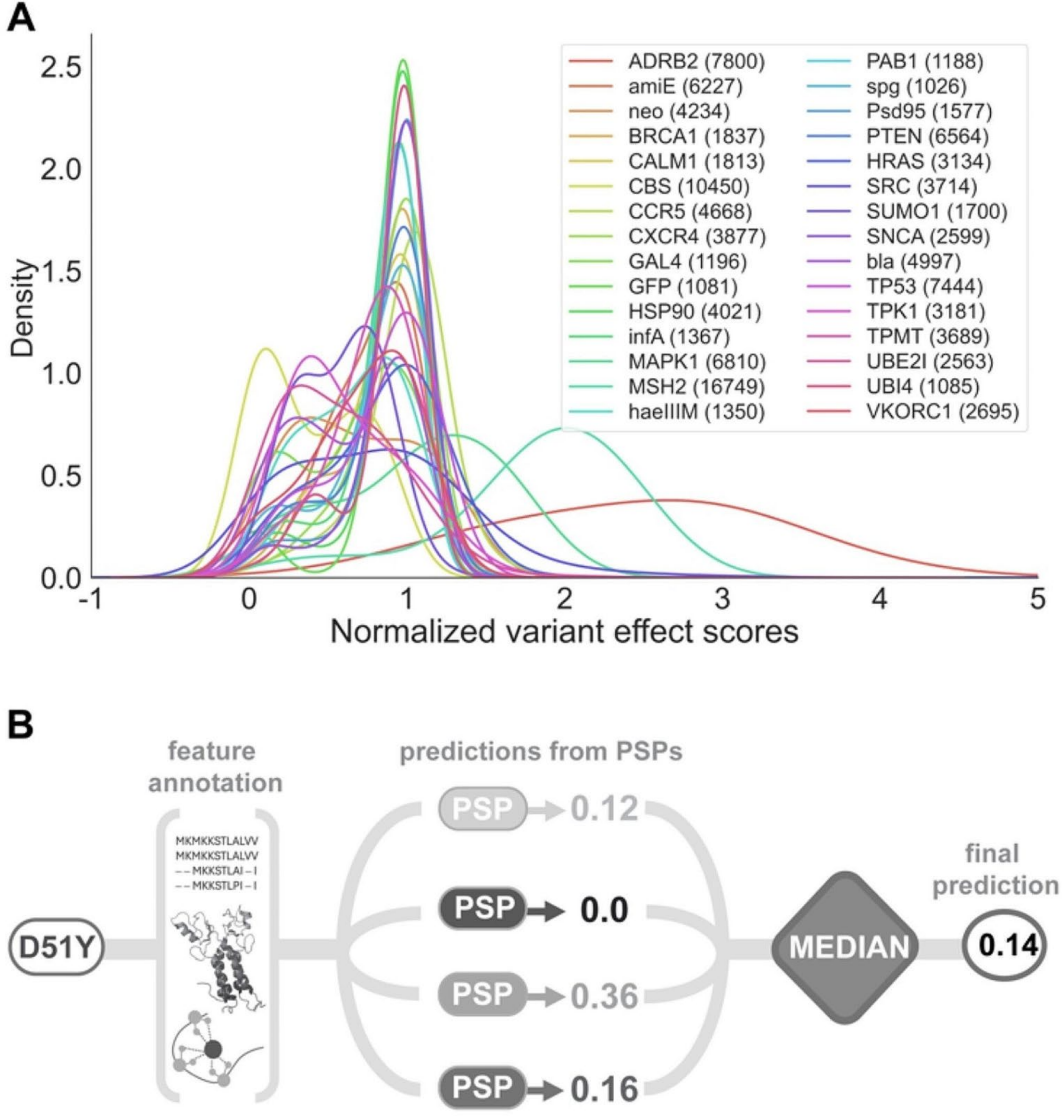
The Quantitative Impact of Missense Variants and its Prediction with QAFI. **(A)** Deep mutational scanning assays have revealed the continuous nature of variant effects on protein function. The figure displays thirty assays utilized in this study to train our prediction method. **(B)** Overview of the QAFI methodology, highlighting the scoring of variants using ten selected protein-specific predictors (PSP) developed for different proteins. It also illustrates how these scores are integrated using the median to produce the final prediction

In recent years, the field has been gradually moving beyond the initial binary classifications of variants to more precise estimates of their functional impact (Masica and Karchin 2016; Livesey and Marsh 2022; Diaz et al. 2023). This new approach opens the door to a better understanding of variant effects, including crucial aspects like disease severity and patient responses to treatments—factors that are essential for Precision Medicine but are not easily addressed through binary classifications. This paradigm change has been fueled by a mixture of conceptual considerations and recent experimental results. On the experimental side, Deep Mutational Scanning (DMS) experiments (Fowler and Fields 2014) have clearly shown (Fig. 1A) the continuous nature of the functional impact of mutations. In parallel, reflecting on the conceptual foundations of pathogenicity prediction, Massica and Karchin (Masica and Karchin 2016) advocated for a significant methodological shift: employing quantitative measurements related to phenotypes (catalytic activity, cell growth rates, etc.) as the new objective functions in model building processes, moving away from traditional binary labels.

Following this paradigm, various authors have worked on quantitative prediction approaches for specific proteins, e.g., CFTR (Masica et al. 2014), BRCA1/2 (Padilla et al. 2019), CALM1 (Katsonis and Lichtarge 2019), an effort captured in the recent review of the first ten years of the CAGI experiment (Jain et al. 2024a). Recently, Gray et al.‘s work (Gray et al. 2018) has taken a significant step forward by offering a general solution to the quantitative prediction challenge, utilizing a regression model trained on a set of DMS experiments. Their tool, Envision, has achieved Pearson correlation coefficients ranging from 0.38 to 0.69 depending on the gene. Other studies have also tackled the quantitative prediction problem in the last years (Laine et al. 2019; Kim and Kim 2020; Gelman et al. 2021; Luo et al. 2021; Song et al. 2021; Wittmann et al. 2021; Zhang et al. 2022; Diaz et al. 2023; Fu et al. 2023; Livesey and Marsh 2023). While some use supervised methods, others have successfully explored how unsupervised methods align with DMS experiments. Among the latter, the work by Frazer et al. (Frazer et al. 2021) is remarkable because these authors build a deep generative model using evolutionary information that reflects the quantitative impact of variants, showing that it can be used for pathogenicity prediction with promising results. More recently, Cheng et al. (Cheng et al. 2023), again using an unsupervised approach and state-of-the-art AlphaFold (Jumper et al. 2021) structural models have reached competitive results in reproducing DMS experiments. However, despite the advances reported in the field (Livesey and Marsh 2023), a consistent trend has emerged: prediction performance significantly varies across different proteins, and no existing method universally excels for all proteins. Such variability underscores a crucial insight: although progress has been notable, a comprehensive solution that encompasses the entire clinical genome remains elusive. This challenge highlights the inherent complexity of predicting missense variants’ functional impacts and underscores the essential need for novel approaches. These strategies should aim to bring us closer to a solution, whether by offering improvements across the entire clinical genome or only for a subset of genes.

In this study, we present a novel methodology for the **Q**u**A**ntitative estimation of the **F**unctional **I**mpact of missense variants, referred to as QAFI (Fig. 1B), that combines protein-specific regression models within the ensemble learning framework (Bishop 2006). More specifically, multiple linear regression models are used to separately address the quantitative prediction problem for a set of proteins for which DMS experiments are available in the literature. To build these protein-specific models, variants are characterized using conservation- and structure-related features (derived from AlphaFold structure models). In a second step, we use a subset of the resulting protein-specific predictors to build a general prediction algorithm for all variants in any protein. For this part, we apply a basic ensemble Learning principle, conceptually based on the fact that certain effects of variants are universal across proteins and our models inherently account for them. To validate the developed procedure, we employed two independent strategies. Initially, an early QAFI prototype was tested in the CAGI6 contest, focusing on ARSA protein variants. This provided preliminary external validation. Subsequently, QAFI’s final version was applied to a broader set of clinically labeled (pathogenic/benign) variants from proteins outside our training dataset, demonstrating our approach’s generalizability and robust predictive power.

## Methods

### DMS variant datasets

We compiled DMS experiments from five publications (Gray et al. 2018; Riesselman et al. 2018; Reeb et al. 2020; Dunham and Beltrao 2021; Frazer et al. 2021), each of which had curated DMS experiments for predictive purposes. Our selection criteria were: (i) use of only missense variants; (ii) availability of a minimum of 1000 variants per experiment; (iii) presence of an AlphaFold model (Jumper et al. 2021; Varadi et al. 2024). If there were several DMSs available for the same protein, we selected either the most recent dataset or the one with the most comprehensive coverage.

After applying these filters, we established a final dataset comprising thirty DMS experiments for thirty distinct proteins (Supplementary Table S1). To standardize the data across different experiments, we adopted the normalization scheme utilized by Gray et al. (2018) in the development of their quantitative predictor. In this scheme, a score of 1 represents variants with no detectable impact on protein function, according to the experimental assay. Scores below 1 indicate decreased activity, and scores above 1 suggest enhanced activity compared to the wild-type.

The resulting mutation dataset is provided in Supplementary Table S2, where we present the normalized scores for all variants studied in the thirty DMS experiments.

### Clinically labeled variants datasets

To validate QAFI using clinically labeled variants, we utilized two distinct datasets of pathogenic and benign variants. The first dataset was employed to establish the decision threshold for classifying QAFI predictions into these two categories. This dataset was compiled from variants retrieved from the HumSavar (UniProt) (Bateman et al. 2017) and ClinVar (Landrum et al. 2016) databases. For ClinVar, we selected variants labeled as pathogenic, likely pathogenic, pathogenic/likely pathogenic, benign, likely benign, or benign/likely benign. These labels were unified into two classes: variants labeled as benign, likely benign, and benign/likely benign were grouped into the benign category; similarly, labels indicating pathogenicity were grouped into the pathogenic category. Variants with a review status of “no assertion criteria provided” were excluded. From HumSavar, variants labeled as LP/P and LB/B were included in the pathogenic and benign categories, respectively. In cases of discrepancies between ClinVar and HumSavar annotations, ClinVar labels were prioritized. Only variants from proteins with an available AlphaFold model were included. Additionally, proteins contributing fewer than 50 pathogenic and 50 benign variants were excluded.

The second dataset, used for constructing Receiver Operating Characteristic (ROC) curves in the Results section, was derived from the aggregated ClinVar 2019 and 2020 sets used by Pejaver et al. (2022) to calibrate computational tools for clinical applications. Similarly, proteins without AlphaFold models were excluded.

To avoid second circularity issues (Grimm et al. 2015), we ensured that none of the thirty proteins used to train our protein-specific models were included in either dataset. Furthermore, we verified that no protein contributed variants to both the first and second datasets of clinically labeled variants.

After processing, the first dataset comprised a total of 6894 variants (4582 pathogenic and 2312 benign) from 25 proteins, while the Pejaver-based dataset contained 8512 variants (3537 pathogenic and 4975 benign) from 1879 proteins.

### Multiple sequence alignments (MSA)

Some of the features used in our methodology rely on the utilization of MSAs. These MSAs were obtained using a procedure detailed by Riera et al. (2016), which we briefly summarize here for clarity. For each target protein, using the sequence available from UniProt, the following steps were executed. First, homolog Identification: Homologs were identified by searching the UniRef100 database (Suzek et al. 2015) using PsiBlast (Altschul et al. 1997). The search was conducted with an E-value threshold of 0.001 over two iterations. Second, sequence Selection: From the PsiBlast output, homolog sequences exhibiting less than 40% identity (computed from the global sequence alignment) to the target protein were discarded. And third: The remaining sequences, including the query sequence, were aligned using Muscle (Edgar 2004) to produce the final MSA.

### Sequence-based features

To construct our quantitative predictors, we utilized fourteen features, including five sequence-based ones. Three of these—elements of the Blosum62 matrix, Shannon’s entropy, and position-specific scoring matrix (PSSM) at the mutation locus—have been previously utilized in other prediction efforts and are described in detail elsewhere (Riera et al. 2016; Padilla et al. 2019).

The fourth sequence-based feature is the average Shannon’s entropy of sequence neighbors, ranging from − 3 to + 3 around the mutated residue, derived from the MSA of the protein family, excluding the native location itself.

The fifth sequence-based property is termed neighbor compatibility (neco). We devised this parameter to estimate the influence of neighboring residues on the likelihood of a specific substitution of the native residue. It is defined as follows:

$$\:neco=\:ln\left(\frac{p\left(Hom=M\:\right|\:Hs=N,\:\text{n}\text{e}\text{i}\text{g}\text{h}\text{H}\text{s}=\text{n}\text{e}\text{i}\text{g}\text{h}\text{H}\text{o}\text{m})}{p\left(Hom=M\:\right|\:Hs=N)}\right)$$

where $\:p\left(Hom=M\:\right|\:Hs=N,\:\text{n}\text{e}\text{i}\text{g}\text{h}\text{H}\text{s}=\text{n}\text{e}\text{i}\text{g}\text{h}\text{H}\text{o}\text{m})$ is the probability of observing the residue M in a homologous sequence at position i (Hom=M) in a MSA, given that the corresponding human residue at that position is N (Hs = N), and the residues at positions i-1 and i+1 are the same between the human and homolog sequences for each respective position (neighHs = neighHom). The probability p(mut=M | nat=N) is used as a normalization factor, to adjust for the baseline frequency of the homolog residue M in the presence of the human residue N.

The calculated values of this parameter are organized in a 20 × 20 substitution matrix, which is utilized for scoring variants. The native and mutant residues in the variant scoring context correspond to the human and homolog residues, respectively, as defined in the formalism.

The substitution matrix is derived by applying the probabilistic definition to multiple sequence alignments of proteins from a well-curated dataset comprising 593 PDB structures (Wang and Dunbrack 2003).

### Structure-based features

These nine features are derived from structure models generated by (Jumper et al. 2021; Varadi et al. 2024); and several incorporate MSA information. They are intended to capture complementary aspects of the impact of missense variants on protein structure and function. We describe them below.

AlphaFold’s per residue confidence score (pLDDT). The values of this parameter are related to the disordered state of the protein region around the residue (Ruff and Pappu 2021).

Binary confidence score (pLDDTbin). This index, derived from pLDDT, only has two values: 1 (pLDDT $\:\ge\:$ 70) and 0 (pLDDT $\:<$ 70). The remaining structural features are all dependent on pLDDTbin: if pLDDTbin = 0, these features are set to 0. It must be noted that while some AlphaFold predictions have very high confidence (pLDDT > 90), using this higher threshold would significantly reduce the number of variants with available structural information (e.g., for MSH2, from 14,704 to 6,589 variants). Therefore, we chose the lower threshold of 70, which still indicates high confidence predictions.

Contact layer size (colasi). This parameter, which varies between 0 and 1 (except in a few cases of residues buried in highly dense locations), is a coarse-grained measure of the network of atomic interactions around the native residue. Colasi is obtained from the AlphaFold model of the native protein following a two-step procedure. First, compute the number of atomic contacts between the atoms of the native residue and those of other protein residues, with distances ≤ 5 Å. This was done using the software Arpeggio (Jubb et al. 2017). Secondly, divide this number by the maximum number of interatomic contacts observed for the native residue’s type in a well-curated dataset of 593 PDB structures (Wang and Dunbrack 2003).

The next three features provide a complementary view of the conservation degree of the residues around the native residue, using Shannon’s entropy in different ways.

Fraction of conserved 3D neighbors. To calculate this parameter, our starting point was the set of atomic contacts of the native residue obtained for colasi (see above). The fraction of conserved 3D neighbors is equal to the number of contacts involving atoms from highly conserved neighboring residues (Shannon’s entropy < 1.37) divided by the total number of contacts. The Shannon’s entropy threshold chosen, 1.37 (one third of 4.12, Shannon’s entropy maximum value), is defined to ensure that most residues engaged in packing networks of functional significance, including protein-protein interactions and the protein core, are included.

The next two features (Fanc and Fbnc: fraction of accessible and buried neighbors conserved, respectively) take into account the accessibility of the native’s neighbors; they are computed as follows. First, we obtain, for the protein of interest, the medians m_acc_ and m_bur_ of the Shannon’s Entropy for the accessible (colasi ≤ 0.5) and buried (colasi > 0.5) residues, respectively. Second, we divide the list of the native’s residue contacts into two categories: accessible and buried. Third, for Fanc, we identify the accessible neighbors of the native residue, count how many of them have Shannon’s Entropy below m_acc_, and divide the number of atomic contacts with the native contributed by these residues by the total number of native contacts. Fbnc is computed similarly, using m_bur_ instead of m_acc_.

Miyazawa-Jernigan potential. This potential (Miyazawa and Jernigan 1996) is based on a probabilistic model that captures the predisposition of the 20 natural amino acids to contact each other. It is embodied in a 20 × 20 table and we use it as follows. First, we identify the neighboring residues whose average side chain atom lies within a distance of < 6.5 Å from that of the native residue. For glycines we employ the C-alpha atom for the distance computations. Subsequently, we calculate the difference in contact energies between the sums of native-neighbour and mutant-neighbour interactions, using the upper triangle of the 20 × 20 table provided by Miyazawa and Jernigan (Miyazawa and Jernigan 1996), as follows:

$$\:{\varDelta\:e}_{mut,nat}=\:{\sum\:}_{j}({e}_{mut,j}-\:{e}_{nat,j})$$

where $\:j$ goes through the list of neighbors; $\:mut$ and *nat* refer to the mutant and native residues, and *e*_*mut, j*_ and *e*_*nat, j*_ are the values of the corresponding Miyazawa and Jernigan table element associated to the pairs of contacting residues (e.g., *e*_*mut, j*_ is the interaction energy between the *mut* residue and the neighboring residue *j-th*). Note that we assume that the neighbors list does not change between human and mutant. This assumption is justified by the step-wise nature of the potential.

Accessibility dependent volume term. This term is designed to reflect packing disruptions arising from size differences between amino acids, weighting them by colasi, to consider environment differences. The formula is equal to:

*Acc. dependent volume* = Δ*size*$\:.colasi$

where Δ*size*=$\:{(\text{max}nc}_{mut}-\:{\text{max}nc}_{nat})$ and $\:{max\:nc}_{mut}$ is the maximum number of contacts the mutant can have (in our curated PDB dataset, see the description of colasi above) and $\:{\text{max}nc}_{nat}$ is the same for the native. These two terms are related to the amino acid volumes of each residue.

Likelihood of the accessibility state for a given amino acid replacement (laar). This parameter is based on a probabilistic formalism similar to that of neco. This parameter is designed to estimate the influence of the accessibility state at the native locus on the likelihood of a specific substitution of the native residue. It is equal to:

$$\:laar=\:ln\left(\frac{p\left(Hom=M\:\right|\:Hs=N,\:Acc=X)}{p\left(Hom=M\:\right|\:Hs=N)}\right)$$

where $\:p\left(Hom=M\:\right|\:Hs=N,\:Acc=X)$ is the probability of observing the residue M in a homologous sequence at position i (Hom=M) in a MSA, given that the corresponding human residue at that position is N (Hs = N), and its accessibility state is X (Acc = X). Only two accessibility states are considered in this computation: buried (colasi > 0.5) and exposed (colasi $\:\le\:$0.5). The probability p(mut=M | nat=N) is used as a normalization factor, to adjust for the baseline frequency of the homolog residue M in the presence of the human residue N.

The calculated values of this parameter are organized into two distinct 20 × 20 substitution matrices: one for buried residues and one for exposed residues. These matrices are used for scoring variants and are analogous to environment-specific substitution tables in threading methods (Shi et al. 2001). In this scoring system, the native and mutant residues correspond to the human and homolog residues, respectively.

The substitution matrices are derived by applying the probabilistic definition to multiple sequence alignments of proteins from a well-curated dataset comprising 593 PDB structures (Wang and Dunbrack 2003).

Both sequence and structure-based features were normalized during training of the model, to transform their range to [0,1] using their minimum and maximum values in the training dataset. The feature values for all the variants in the DMS experiments are provided in the Supplementary Table 2.

### Multiple linear regression (MLR) models

For our protein-specific predictors, we employ standard MLR models, which assume a linear relationship between the dependent variable and multiple independent variables. We constructed separate MLR models for each protein, using DMS experiment values as the dependent variable and the 14 derived features as independent variables.

Due to the generally bimodal and often imbalanced distribution of values in DMS datasets (Fig. 1A), which can bias the feature weights in regression models (Torgo et al. 2015), we implemented a two-step undersampling procedure to balance the training data. First, we fitted a Gaussian Mixture Model to identify an optimal threshold separating the two peaks in each dataset. Second, we equalized the number of data points from each distribution peak by randomly reducing the size of the larger group to match that of the smaller one. This balancing was applied only to the training set during the cross-validation process.

For cross-validation, we used a stringent variant of Leave-One-Out Cross-Validation (Porras et al. 2024), where all variants at a single position are held out as a test set, while those at other positions form the training set. This process is repeated until each position has been excluded once, ensuring that all data points serve both as training and testing data at different stages.

The MLR models were developed using the scikit-learn package in Python (Pedregosa et al. 2011).

### Selection of the ten protein-specific predictors for the final version of QAFI

In QAFI, ten protein-specific predictors are central to the prediction procedure. We selected these ten from the original set of thirty protein-specific predictors, which were developed for each protein in our DMS dataset. To determine the ten most effective predictors, we employed a specialized leave-one-out cross-validation procedure (Supplementary Fig. S1). In this approach, for each round of validation, one protein was removed from the dataset, and the performance of the predictors was evaluated on the remaining proteins. We recorded the performance of each predictor in terms of their ability to accurately predict variant impacts on the other proteins. The ten predictors that most frequently showed the highest performance across these rounds were selected for the final version of QAFI.

### Obtaining a threshold for the binary (pathogenic/benign) classification of variants from QAFI predictions

To establish a threshold for classifying variants as pathogenic or benign based on QAFI quantitative predictions, we utilized a dataset of 6894 clinically annotated variants across 25 proteins with more than 50 pathogenic and 50 benign variants (see above). For each protein, we calculated the QAFI predictions for its variants. We then systematically explored a range of potential threshold values from 0 to 1.5. For each threshold value in this range, we computed the Matthews Correlation Coefficient (MCC) for the classification performance. After pooling the MCC values from all proteins, we selected the median MCC at each threshold. The optimal threshold value, which corresponded to the highest median MCC, was determined to be 0.82.

### Binary classifiers for comparative analysis

To benchmark our binary predictions, we selected 13 in silico tools whose clinical applicability was recently evaluated by Pejaver et al. (2022). These tools include REVEL (Ioannidis et al. 2016), BayesDel (Feng 2017), VEST4 (Carter et al. 2013), MutPred2 (Pejaver et al. 2020), CADD (Rentzsch et al. 2019), EA (Katsonis and Lichtarge 2017), SIFT (Kumar et al. 2009), PolyPhen2 (Adzhubei et al. 2010), MPC (Samocha et al. 2017), PrimateAI (Sundaram et al. 2018), GERP++ (Dong et al. 2015), FATHMM (Shihab et al. 2013), and PhyloP (Pollard et al. 2010). Predictions from these tools for the clinically labeled variant dataset were obtained from the study by Pejaver et al. (2022). Additionally, we incorporated predictions from the methods EVE (Frazer et al. 2021) and AlphaMissense (Cheng et al. 2023), and Envision (Gray et al. 2018), which were accessed directly from their respective websites.

### Writing support

The text of this article was reviewed for grammar and clarity using ChatGPT, an AI language model. All corrections suggested by ChatGPT were carefully reviewed to ensure they accurately reflect our intended results.

## Results

This section is divided into two main parts. Initially, we detail the development of QAFI, including the creation of protein-specific predictors and the application of ensemble learning principles to establish a method applicable across the entire clinical genome (Fig. 1B). Subsequently, we present the validation of our methodology through two distinct approaches: participation in the CAGI6 ARSA challenge and evaluation using a comprehensive dataset of pathogenic and benign variants from various genes.

### QAFI development

Our method was formulated in two stages. Initially, we tackled the quantitative prediction of the functional impact of variants for a set of thirty proteins, for which DMS experiment data were available. This led to the development of thirty protein-specific predictors. Following this, we utilized an ensemble learning-based strategy to extrapolate our predictions to any variant within any protein, leveraging the results of the protein-specific predictors.

#### Constructing and training thirty protein-specific models using DMS Data

After reviewing the literature, we compiled a dataset comprising thirty human proteins (see Supplementary Table S1), selected based on the availability of DMS experiments with over 1000 variants each. This threshold was established to ensure a sufficient amount of variants for the construction of independent, protein-specific, MLR models. We chose MLR for its simplicity and interpretability.

Each MLR model incorporated fourteen features (Methods Section) designed to quantify the impact of variants. These descriptors integrate sequence and structure information (derived from AlphaFold models). They include metrics that capture the influence of residues close to the native residue —either in sequence or spatially—on changes occurring at the mutation site.

Finally, we determined the model’s parameters for each protein by fitting them to the DMS experimental data specific to that protein. Since the effect of variants happening at the same position is not independent (Cheng et al. 2023), to evaluate the accuracy of the resulting models we adopted a rigorous version of the Leave-One-Out Cross-Validation strategy (Porras et al. 2024). In this approach, all variants at a given position are exclusively assigned to either the training set or the validation set in any cross-validation round. A list of the full variant dataset, including normalized functional values and predictions from both protein-specific models and QAFI, is provided in Supplementary Table S2.

**Fig. 2:**
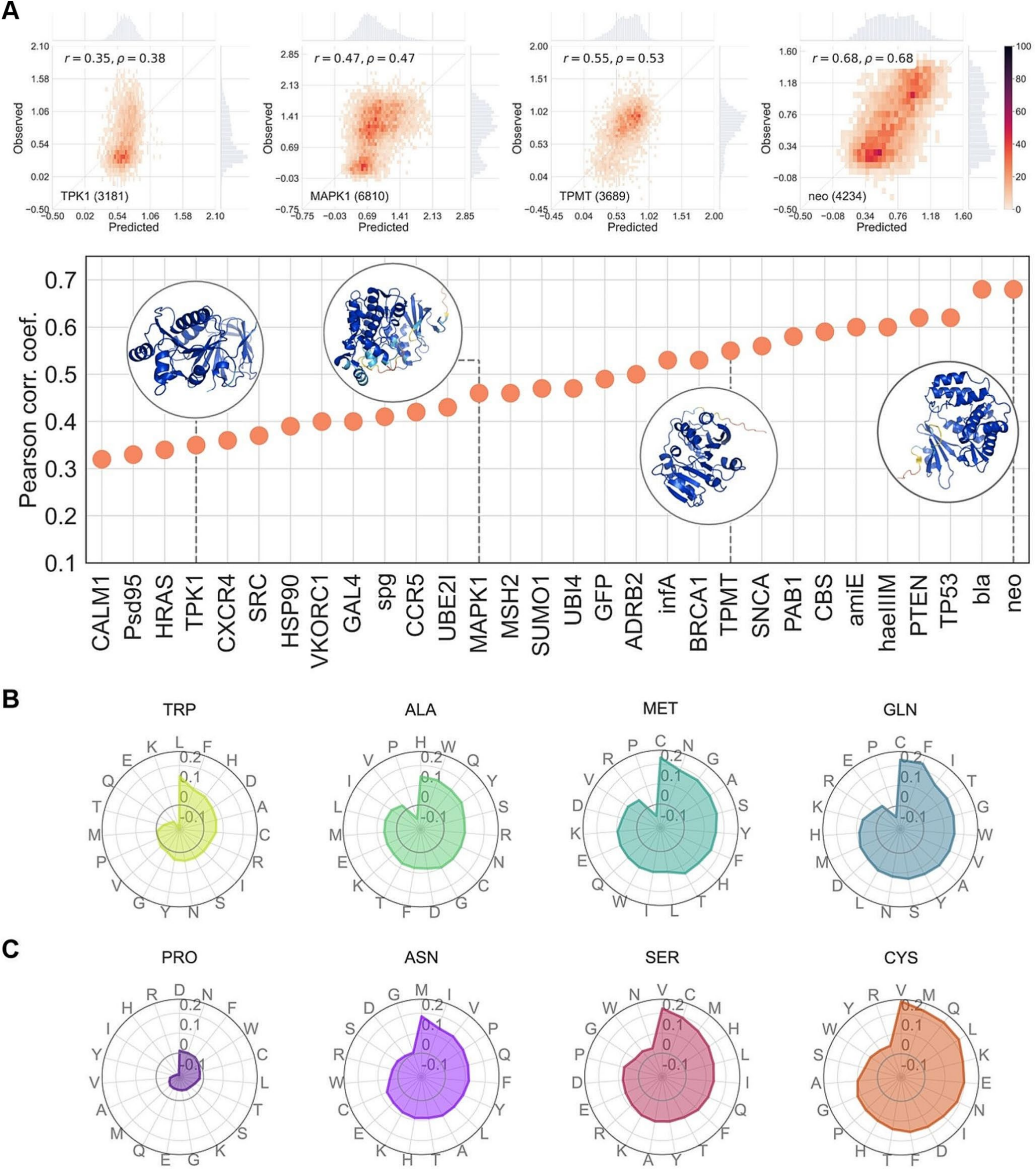
Performance of Thirty Protein-Specific Predictors Developed Using MLR. **(A)** Display of position-cross-validated Pearson correlation coefficients for auto-prediction, where each model is applied to variants from its corresponding protein. Circles indicate four selected proteins, chosen to represent a range of prediction accuracies. Above, heatmaps show the observed vs. predicted plots for these proteins. **(B)** Median prediction error for four native amino acids; radar plots illustrate the challenge of predicting their nineteen possible mutations. **(C)** Median prediction error for four mutant amino acids; radar plots show the difficulty in predicting variant impacts for each of these amino acids as the mutant residue. For B and C, an expanded plot covering all twenty natural amino acids is provided in Supplementary Fig. S2

In Fig. 2A, we display the observed vs. predicted comparison for the resulting protein-specific models. The figure shows that the predictive accuracy for the different proteins, as measured by Pearson correlation coefficients, varies within a range of 0.3 to 0.7 (see also Supplementary Table S3). Above the figure, we present four examples that illustrate the correspondence between the values of the Pearson correlation coefficients and the explicit Observed vs. Predicted comparison. As expected, we see increasingly clear linear behavior for the latter as correlations increase.

Beyond the protein-level performance, we also explored the per-residue performances. This analysis is of interest because it helps us determine whether the features used are equally effective for all residues, both native and mutant. This insight is crucial for guiding future improvements of our method and is relevant from the perspective of users of our methodology. We separately analyzed the native and mutant cases. In Fig. 2B, and Supplementary Fig. S2, we present the median deviation in our predictions for each of the twenty natural amino acids when they serve as the native residue of a variant. In Fig. 2C, and Supplementary Fig. S3, we provide a similar plot focusing on the mutant residues. The findings are consistent across both contexts: some residues yield less accurate predictions when they are native, and a similar pattern is observed for mutant residues. For example, predictions for variants where the native residue is a tryptophan are easier to predict, in general, than when it is a glutamine (Fig. 2B); or predictions when the mutant residue is a cysteine are harder to predict than when it is a proline (Fig. 2C).

#### QAFI: generalizing variant impact predictions to the Proteome using ensemble learning

**Fig. 3:**
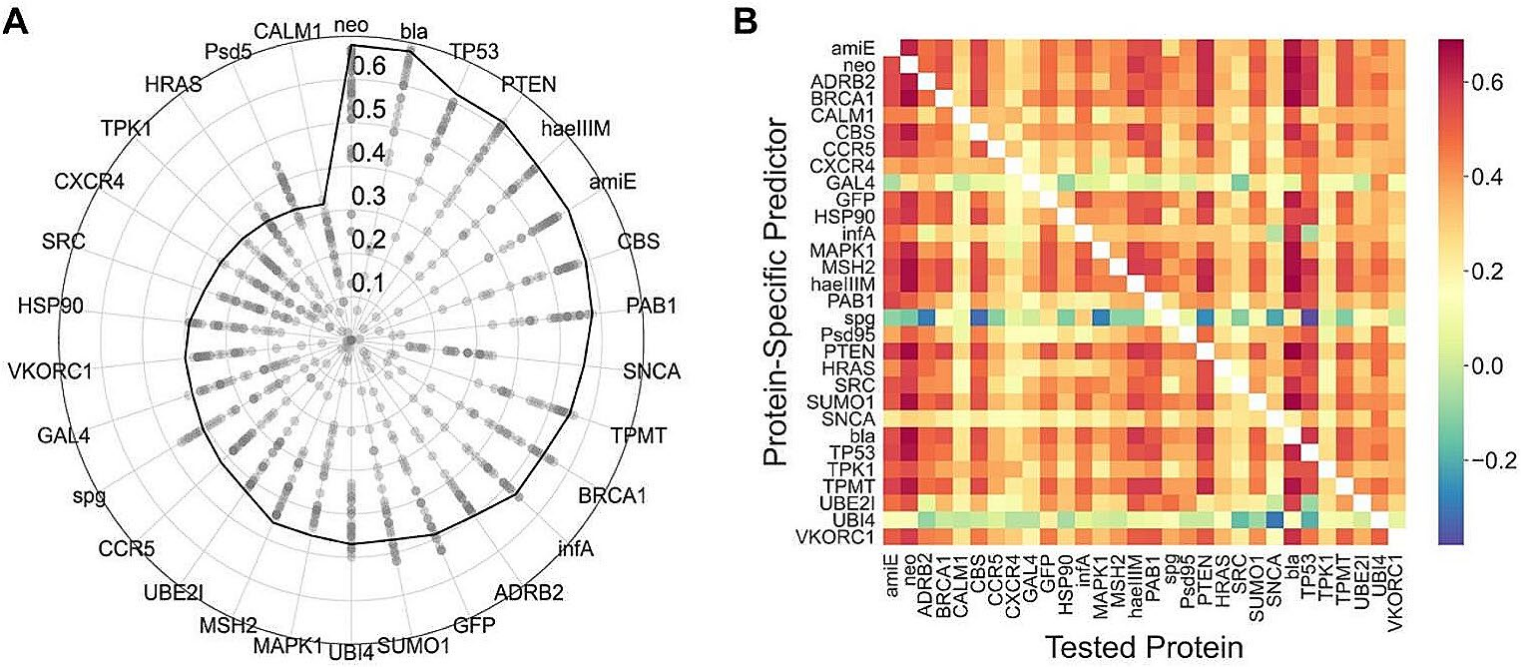
Cross-prediction experiment. **(A)** Radar plot displaying the results of the cross-prediction experiment for each of the thirty proteins, where predictions for each protein’s variants are generated by applying the protein-specific models of the other twenty-nine proteins. The scale of the radii corresponds to the Pearson correlation coefficients. The continuous line indicates the result from the auto-prediction experiments shown in Fig. 2A. **(B)** Heatmap detailing the effectiveness of each protein-specific predictor (vertical axis) in estimating impacts across different proteins (horizontal axis), with color coding reflecting correlation values. Diagonal cells representing auto-predictions are left white to prevent confusion

First, we evaluated the capacity of protein-specific predictors to generate quantitative impact estimates for variants in proteins different from those used in their training. We will refer to these estimates as cross-predictions, in contrast with the auto-predictions, which are the estimates obtained with the protein-specific predictor trained on data for the same protein (Fig. 2A). The radar plot in Fig. 3A reveals that although cross-predictions generally offer less precise impact estimates compared to auto-predictions, there are consistently models for almost all proteins that perform comparably (e.g., for TEM1, PTEN, etc.) or, in some instances, exceed (e.g., for PSD95 or Protein G) the performance of the auto-predictor. This finding is further detailed in the heatmap of Fig. 3B, which highlights the variance among predictors. For instance, the PTEN row displays predominantly reddish cells, indicating higher Pearson correlations in the cross-predictions of this model, whereas the SNCA row, with predominating yellowish cells, indicates a lack of cross-prediction accuracy.

Analogous to what occurs in the classification version of the problem (Riera et al. 2016; Livesey and Marsh 2023), the results in Fig. 3 indicate that, for our problem, protein-specific models can identify components of the quantitative impact of variants across proteins, as if they were general predictors. In this situation, it is natural to consider the use of ensemble learning approaches, combining the outcomes of various tools (Bishop 2006), to enhance predictive performance and extend our methodology to proteins other than the 30 proteins in our original dataset. Here, we apply this idea and suggest that the median of cross-predictions for a given variant effectively represents its impact on protein function. This approach constitutes the core of our method, QAFI.

**Fig. 4:**
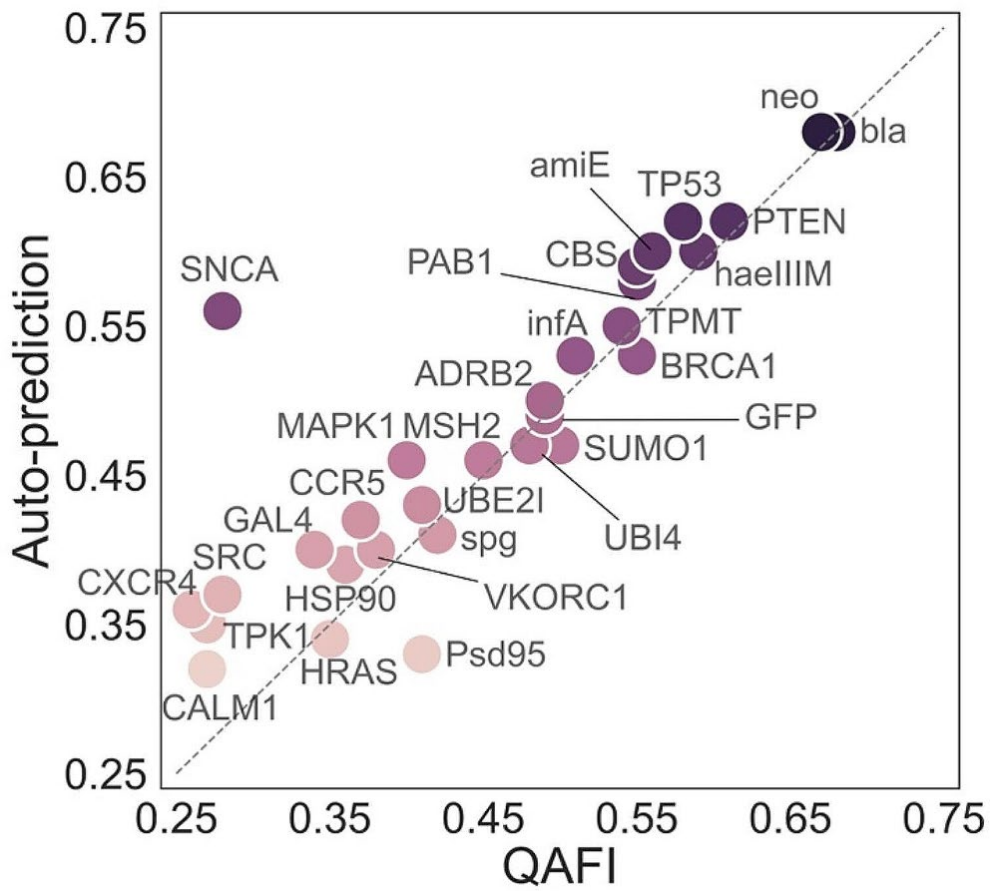
Performance Comparison Between Protein-Specific Predictors and QAFI. This figure compares the auto-prediction results of protein-specific predictors (Fig. 2A) with those from QAFI, for the dataset of thirty proteins. For the ten proteins that contribute a predictor to QAFI, a modified version of this tool was used, excluding the predictor corresponding to the protein being tested from the median computation (Fig. 1B). Both axes represent Pearson correlation coefficients

Technically, since some predictors clearly outperformed others in the cross-prediction experiment, and some showed notably poor performance (Fig. 3B), the current version of QAFI specifically incorporates the top ten performing models from these experiments. These ten predictors were obtained following a specialized leave-one-out cross-validation procedure (Supplementary Fig. S1). In each validation round, one protein was omitted from the dataset, and the performance (Pearson correlation coefficient) of the protein-specific predictors on the remaining twenty-nine proteins was assessed, excluding their respective training protein. These predictors were then ranked based on the median of their Pearson correlations. The rank of each predictor was recorded over the twenty-nine rounds in which it was part of the training set. The ten predictors most consistently demonstrating the highest performance were selected for the final version of QAFI: PTEN, haeIIIM, MSH2, neo, TP53, TPMT, ADRB2, bla, SUMO1, and amiE. Note that in each round, the performance of the QAFI version applied to the left-out protein was used to produce the cross-validated results shown in Fig. 4.

It must be emphasized that after the selection of the ten methods, the development of QAFI is finished; that is, no additional parameter training step was applied. For a given variant from any protein, the QAFI score is obtained taking the median of the ten predictors’ scores for that variant (Fig. 1B), which is an entirely non-parametric procedure.

#### Performance and feature analysis of the protein-specific predictors

In this section, we address two aspects of our models’ performance: the factors contributing to this performance and the role of predictive features in the model.

To understand the components of the predictive performance of the selected protein-specific models, we have focused on three key aspects related to the predictive power of regression models: data-to-parameter ratios, compositional diversity of the training samples, and the behavior of both response and explanatory variables. For each of these factors, we compared the behavior of the ten chosen proteins with that of the twenty remaining proteins.

Data-to-Parameter Ratios. Considering that all our regression models have the same number of parameters (14), we simplified our analysis by focusing on the amount of data, i.e., the number of variants, contributed by each protein. In general (Supplementary Fig. S4A), the ten chosen proteins contribute more variants than the twenty remaining proteins. Specifically, eight of the ten proteins had a large number of variants (well over 3500), whereas only six of the remaining twenty proteins surpassed this limit. Only two out of the ten proteins (haeIIIM and SUMO1, with 1350 and 1700 variants, respectively) had variant numbers closer to the lower threshold we set to ensure model quality. Given that good data-to-parameter ratios contribute to better parameter estimates, we believe that higher model robustness might be a factor in determining the final list of ten proteins.

Compositional Diversity. We analyzed the amino acid composition of the wild-type sequences (Supplementary Fig. S4B), finding it comparable between the ten and the twenty proteins’ datasets. We also studied the number of mutations per position (Supplementary Fig. S4C). The median number of mutations per position is 19 for three of the ten proteins, above 15 for six, and only low (< 5) for haeIIIM. This ensures good sampling of the mutation space for most of the ten selected proteins, which is important for applying their specific predictors to other proteins.

Response (normalized scores) and explanatory variables (sequence and structure-based features) of the regression models. First, we compared the normalized functional scores (Supplementary Fig. S4D), noting that, except for two proteins, the scores for the ten proteins cover a range similar to those of the other twenty proteins. Next, we turned our attention to the explanatory variables, focusing on the value distributions of the five most discriminant properties (Supplementary Fig. S4E; these properties are the five top ranking ones in Supplementary Fig. 5A), finding a clear overlap between the ten chosen proteins and the remaining twenty, indicating similar behavior. This is coherent with the fact that, for the ten proteins, the structure of the regression problem is representative of that in the remaining proteins.

In summary, the previous analyses support the idea that the higher performance of the ten chosen predictors results from several factors, including parameter robustness and the fact that the prediction problem for the ten proteins is representative of the same problem in other proteins.

 To understand the relative contribution or weight of each feature to the models’ predictive capacities, we performed a Lasso regression analysis. More concretely, for each protein, we trained Lasso models using a grid search over a range of alpha values from 10^− 5^ to 10^2^. A 10-fold cross-validation scheme was employed to select the optimal alpha value based on the lowest mean absolute error. For the regression models associated with this optimal alpha, we collected the absolute values of each feature’s weights across the thirty proteins. The resulting distribution was plotted using boxplots (Supplementary Fig. S5A), and two specific aspects deserve mention. First, if we focus on the median of the different boxplots, we see that the contribution of the features clearly varies. Interestingly, in the top-ranking positions, we find both 3D and sequence-based features, such as Miyazawa-Jernigan potential (3D), Shannon’s entropy (seq.), colasi (3D), and Blosum62 matrix (seq.). These features tend to be the best predictors across proteins, although their ranking may vary (see below). The presence of the Miyazawa-Jernigan potential is interesting because it goes beyond the geometric description of a residue’s environment and is related to the impact of variants on protein stability (Miyazawa and Jernigan 1996). In a less prominent but also important position, we find neco, which captures the strength of the relationship between the native site and its immediate sequence neighbors. Overall, this analysis highlights the complementary value of sequence and structural information.

A second aspect worth noting is the range overlap in the boxplots in Supplementary Fig. 5A, which indicates that beyond the general trend just mentioned, the relevance of predictive features may change between proteins. This change may, for example, affect Shannon’s entropy and colasi, Miyazawa-Jernigan potential and Blosum62 matrix, among others.

Finally, we focused the Lasso analysis on the ten proteins selected for QAFI (Supplementary Fig. S5B). We see that, apart from the trends mentioned above, there are two aspects of interest. First, the median values of the weights tend to be higher than for the thirty proteins, and second, the boxplot range is tighter. Regarding the latter, although we cannot discard a sampling effect, we see that the ten chosen models appear to be closer in the model space, which may be due to the factors mentioned above (more samples to estimate the parameters, and a larger coherence in the prediction problem). The fact that the boxplots for the general proteins are closer to zero may indicate that our model is less adequate for them, or has not been derived using enough data.

#### Leave-one-protein-out cross-validation of QAFI

To provide an initial assessment of QAFI’s performance, we compared it against the auto-predictions for the same set of thirty proteins (Fig. 4). It should be noted that for the ten proteins whose protein-specific predictors are included in QAFI, the performance measurements were obtained after excluding the corresponding protein from the median computation. This step was taken to prevent potential data leakage that could artificially inflate QAFI’s performance. The figure presents a comparison between QAFI predictions and auto-predictions for thirty proteins, showing a generally good correspondence across the correlation range. Most data points are clustered near or along the diagonal, indicating that QAFI essentially retains the predictive ability of the protein-specific models. However, a small cluster of proteins, such as CXCR4 and TPK1, shows deviations at the lower end of the correlation spectrum. This discrepancy may arise because the features used in our model may not adequately capture the functional effects of variants on these proteins, or the model used by the top ten predictors may not align with the underlying patterns of proteins with lower correlations. Additionally, we identified a clear outlier, the SNCA protein, whose behavior is likely influenced by the presence of hexameric repetitive patterns in its sequence (Sarchione et al. 2021). These patterns could lead to model overfitting, despite rigorous cross-validation.

We compared QAFI’s performance to that of Envision (Gray et al. 2018), EVE (Frazer et al. 2021), AlphaMissense (Cheng et al. 2023), and ESM1b (Brandes et al. 2023). To this end, we selected a subset of fourteen proteins for which at least three of the methods (excluding QAFI) provided predictions. The variant predictions for these tools were downloaded from their respective websites. We chose Envision because it is a reference in the field and represents a careful implementation of the idea of using DMS assays as prediction targets rather than datasets of binary labelled variants. The remaining methods, while not specifically trained to reproduce DMS values show good correlations with them. EVE was identified as a top performer in a recent and extensive benchmark study (Livesey and Marsh 2023) using recently published DMS experiments and a vast array of methods, both binary classifiers and quantitative predictors. ESM1b was also selected for its performance: it outperforms ESM-1v—a top-ranking method assessed in the Livesey and Marsh 2023 benchmark. Finally, we included AlphaMissense, which, leveraging technology derived from the groundbreaking AlphaFold structure prediction method (Jumper et al. 2021), outperforms both EVE and ESM1b. This selection allows us to evaluate our progress relative to Envision and establish our position among the top performers in the field. For this comparison, we employed two standard measures: Pearson correlation coefficient and Mean Absolute Error (MAE). These metrics provide complementary views of how close we are to achieving our prediction goal. The Pearson correlation coefficient assesses the overall relationship between predicted and observed values, indicating how well the predictions align with the actual data trend. Meanwhile, the MAE quantifies the average deviation from observed values and aligns with our objective of providing precise estimates of functional impact, useful for the intended applications. The performance of methods like AlphaMissense, which produce scores between 0 and 1, serves as a baseline benchmark for our method. Ideally, our approach should outperform these bounded-score methods, as their upper-bound value does not accurately reflect the reality of experimental assays (Fig. 1A).

**Fig. 5:**
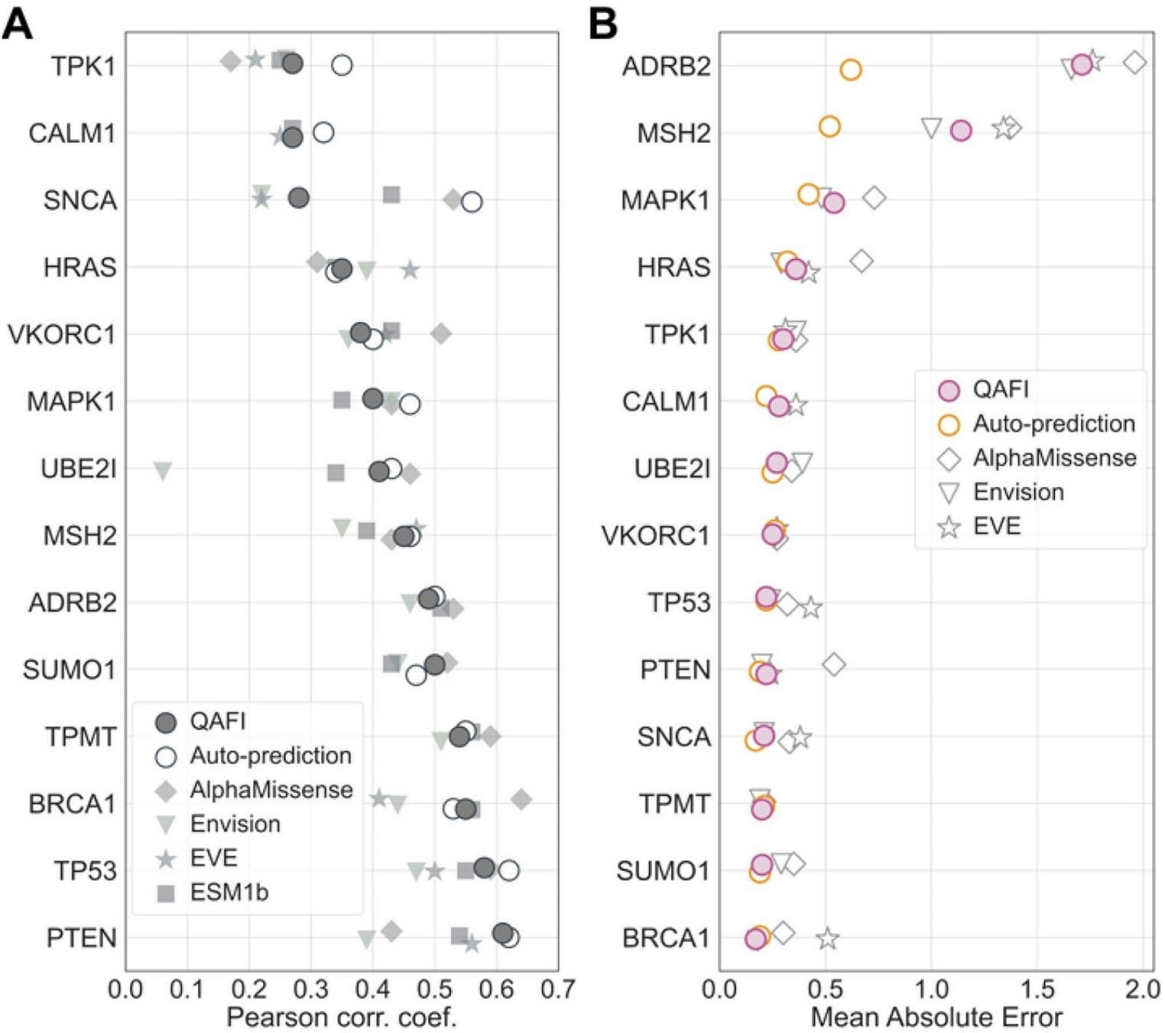
Benchmark of QAFI and Protein-Specific Predictors Against Four Selected Methods. **(A)** Displays Pearson correlation coefficients for each method. **(B)** Shows Mean Absolute Error (MAE) for each method, except for ESM1b, which is excluded due to its prediction scale differing significantly from the observed values, causing a highly compressed figure. In both A and B, proteins are sorted from poorest (top) to best (bottom) predicted according to each parameter

The results obtained (Fig. 5 and Supplementary Table S4) demonstrate consistent behavior across essentially all methods. For the Pearson correlation coefficient (Fig. 5A), some proteins (e.g., TPK1 and CALM1) show universally lower values across all methods, indicating a consistent challenge in predicting their impacts. Generally, our protein-specific predictor performs in the higher range, validating the predictive value of the selected features and regression model. Although QAFI’s performance is slightly lower, it follows the general trend and outperforms Envision. In several cases, it also surpasses ESM1b, EVE, and AlphaMissense.

In terms of MAE (Fig. 5B), the scenario reflects a shift from the Pearson correlation analysis; while overall performance still varies by protein, QAFI and the protein-specific predictors consistently exhibit superior performance, recording the lowest MAEs across almost all proteins. As expected, QAFI’s performance is lower than that of the protein-specific tools, particularly for MSH2 and ADRB2, which are among the most challenging proteins to predict. This underscores its robust ability to reproduce experimental values, maintaining its top-tier status in most cases. Envision deserves a special mention as it outperforms QAFI for several proteins, though the differences are generally minor (e.g., for RAS and MAPK1).

QAFI emerges from these analyses as a tool that can competitively predict the functional impact of variants, demonstrating its efficacy across a diverse array of proteins, although results may vary based on the specific characteristics and challenges associated with each protein.

### QAFI independent validation

To further characterize the performance of our method, we validated QAFI through our participation in the CAGI6 ARSA challenge, and by testing it on a large dataset of clinically labeled variants.

#### Participation in the CAGI6 ARSA challenge

The international CAGI competition allows participants to explore the performance of their technology in a blind application on a set of variants proposed by different independent groups (Jain et al. 2024a). Once the submission process is closed, the experimental results for these variants are made public, and concurrently, a team of assessors evaluates the results for the different groups. A particularly interesting feature of CAGI is that it permits groups to submit up to six prediction proposals, enabling authors to explore multiple versions of their methodologies. However, for assessment purposes, authors must designate which version they consider the most effective.

In our case, when the CAGI 6 ARSA challenge (blind scoring of missense variants in the ARSA protein) was announced, our method was not in its final version as we present in this article. We had a prototype trained with ten features only. For the six different versions, we decided to explore a problem of interest, which is generating meta-predictors from our method. To do this, we used six combinations of QAFI with methods whose performance had seemed good in the analyses we had performed so far. Two of them (Models 2 and 3, see below) involved the use of a simple Random Forest classifier obtained without a hyperparameter tuning step. Based on our group’s experience with similar problems, we utilized the following parameters: a maximum depth of 75, 50 estimators, a minimum of 3 samples per leaf, and 4 samples per split. Additionally, we tested an extended version of QAFI (Models 4 and 6, see below), where the median computation was done using the results from all thirty protein-specific predictors instead of just the chosen ten. The six models were:

**Table:** 

Model 1:	(QAFIMeta): QAFI + REVEL + Envision + EVE
Model 2:	QAFI + RandomForest + REVEL + Envision + EVE
Model 3:	RandomForest
Model 4:	Model 6 + REVEL + Envision + EVE
Model 5:	QAFI (median 10 chosen protein-specific predictors)
Model 6:	Median 30 protein-specific predictors

Models 3, 5, and 6, where used as references for the metapredictors in Models 2, 1, and 4, respectively.

**Fig. 6:**
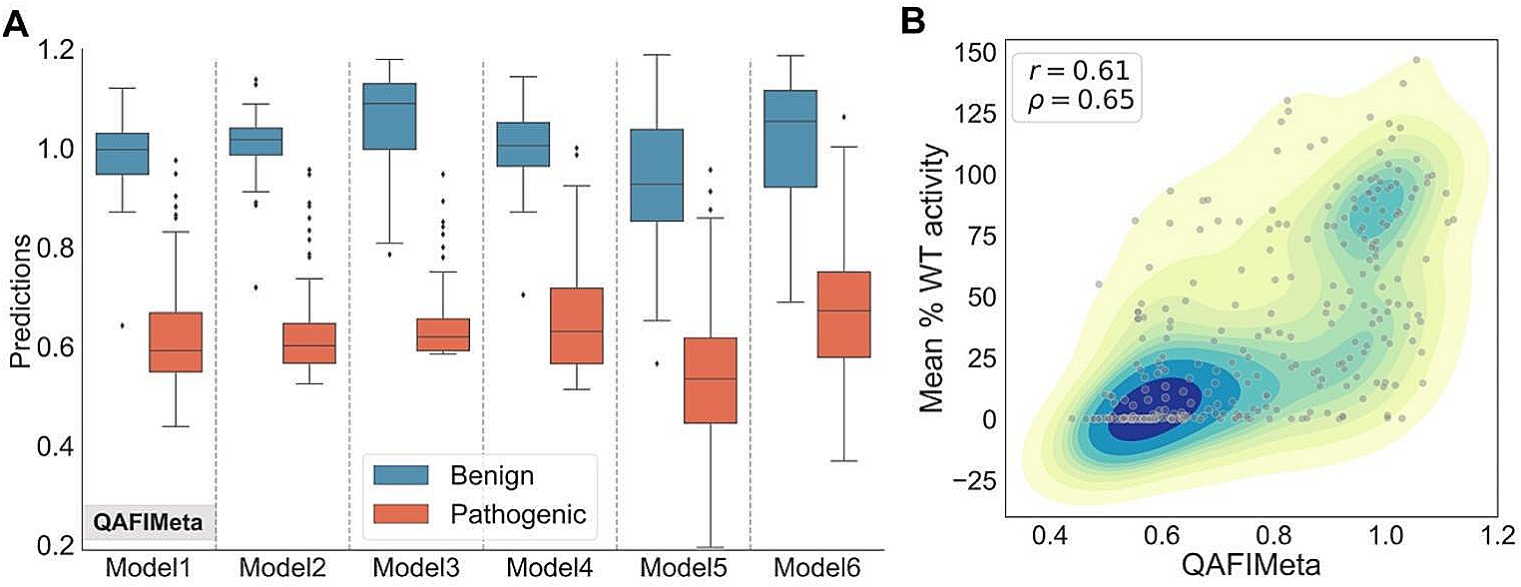
Participation in the CAGI6 ARSA Challenge. **(A)** Boxplots used in the prioritization process for the six models (see Supplementary Table S5) submitted to CAGI6. All models, except for Models 3, 5, and 6, integrate QAFI with predictions from other tools after rescaling their scores to match QAFI’s scale. For two of these models, the QAFI component included all thirty protein-specific predictors for median computation central to QAFI (see Fig. 1B) instead of the standard ten. QAFIMeta was selected as our top candidate, on the basis of its discriminant power and score continuity. **(B)** Comparison of experimental values of mean percentage wild-type activity for ARSA challenge variants (Trinidad et al. 2023) with predictions from QAFIMeta

To select our leading proposal, we used the available data on variants in this protein, data that corresponded to variants whose clinical impact (pathogenic or benign) had been previously described in the literature, and we excluded the few cases that coincided with variants in the CAGI dataset. We then graphically represented the distribution of these variants in relation to the score of our methodology (Fig. 6A) and analyzed them visually, focusing on identifying the method that showed the greatest discrimination power for this set of variants. The chosen method, a combination of QAFI, REVEL, EVE, and Envision, is referred to as QAFIMeta.

For this article, we created an Observed vs. Predicted plot (Fig. 6B) for this version of our method, which showed a Pearson correlation coefficient of 0.61 (see Supplementary Table S5 for the results of the six candidates). This value is in the high range of the previously observed correlation scale (Fig. 2A). The independent evaluation results by the CAGI expert panel (Jain et al. 2024b) showed that QAFIMeta was the second-best performing method (based on a summary of several performance parameters) compared to the other CAGI participants in the ARSA challenge. Notably, when considering R^2^, the most rigorous evaluation measure used by the assessors (and excluded from the final challenge assessment), QAFIMeta ranked first with an R^2^ of 0.252 (see Table S2 from Jain et al. (2024b).

#### QAFI validation using clinically labelled variants

To further validate our methodology, we decided to use data from a problem closely related to ours: the binary classification of missense variants into pathogenic and benign. While the two problems differ in the predictive goal, which in one case is quantitative and in the other categorical, we can take advantage of the fact that molecular impact is a main component of the clinical effect of variants in hereditary disease. In fact, molecular impacts above a certain threshold are typically associated with pathogenic variants; conversely, effects below this threshold generally indicate benign variants. As part of our validation approach, we explored to what extent this correspondence holds for QAFI by discretizing its scores through a threshold and comparing the resulting classes with known clinical annotations. The simplest way to do this is to use ROC curves, as these curves allow testing the effect of different thresholds in the classification of variants. For this study, we used a set of variants created by Pejaver et al. (Pejaver et al. 2022) after a thorough curation process. Additionally, as a reference, we included the ROC curves corresponding to thirteen methods studied by these authors, plus those of Envision, EVE, and AlphaMissense.

**Fig. 7:**
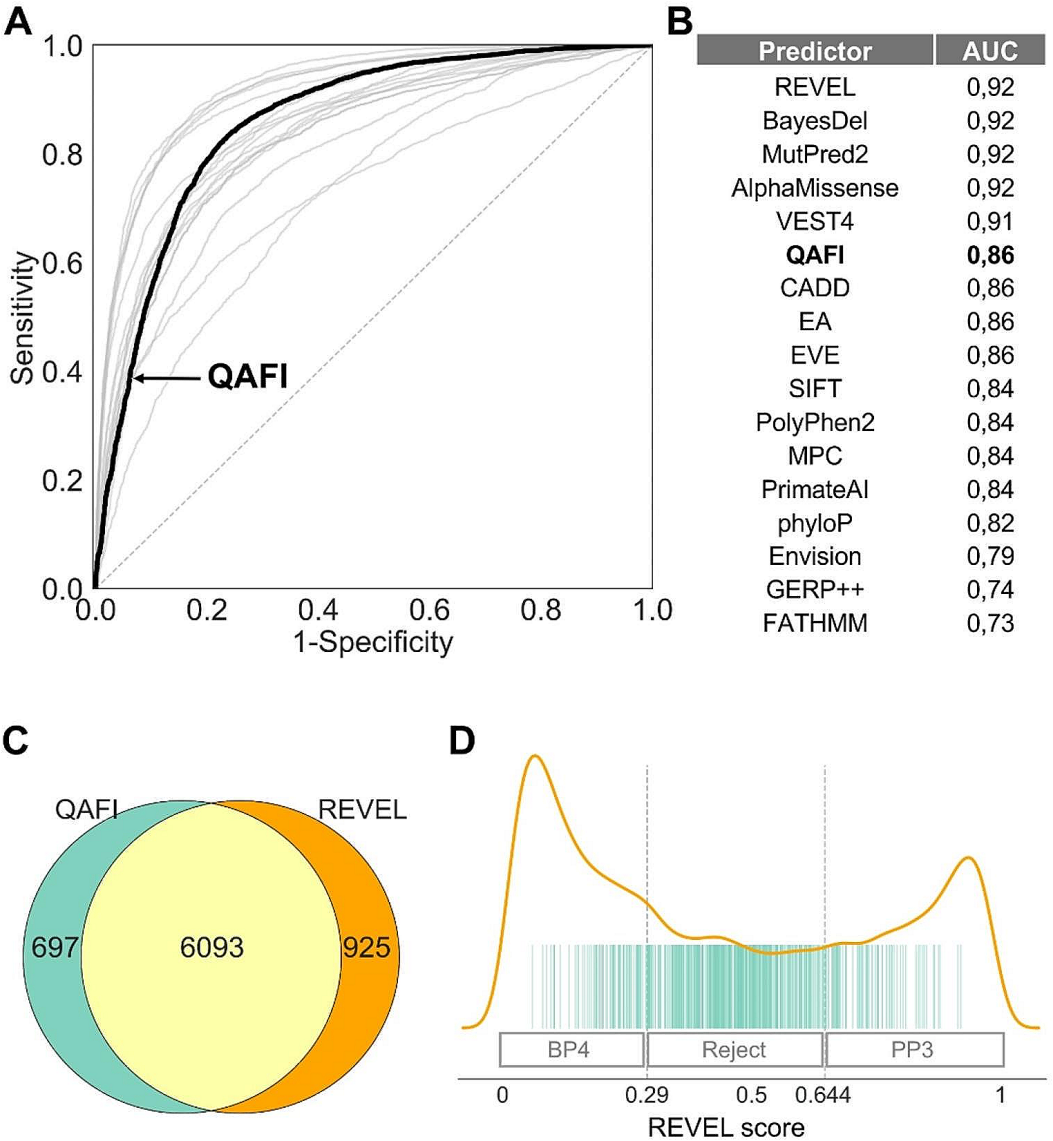
**Validation of QAFI Using Clinically Labeled Variants. (A)** ROC curve displaying QAFI’s performance (black line) compared to sixteen reference predictors (grey lines, see text). **(B)** Area Under the Curve (AUC) values for QAFI and the sixteen other predictors. **(C)** Venn diagram illustrating the complementarity between QAFI and REVEL predictions. Yellow indicates variants correctly predicted by both methods; green and orange highlight variants correctly predicted only by QAFI and REVEL, respectively. **(D)** Distribution of correct predictions by QAFI (green vertical lines) relative to REVEL’s score distribution (orange curve). The boxes above the axis delineate evidence regions for REVEL scores used in clinical annotation of variants, as defined by Pejaver et al. (2022)

In Fig. 7A, we see the ROC curves corresponding to these methods. The first aspect we would like to highlight is that QAFI is significantly distanced from the diagonal line, demonstrating good consistency between its impact predictions and the known clinical effects of the variants, as evidenced by an Area Under the Curve (AUC) of 0.86 (Fig. 7B). The comparison of QAFI with other methods shows that its AUC (Fig. 7B) ranks it on the third position. This is noteworthy because a majority of these classifiers are supervised tools specifically trained on solving the binary classification problem.

To complete the previous analysis, we separately examined whether QAFI successfully predicts variants that are not identified by the top five binary classifiers, thereby underscoring the added value of quantitative approaches. In Fig. 7C and D we present the results of the comparison between QAFI and REVEL (additional results for AlphaMissense, CADD, MutPred2, Vest4, and BayesDel are provided in Supplementary Fig. S6). As illustrated in the Venn diagrams (Fig. 7C), QAFI accurately predicts 697 variants that REVEL does not correctly identify. This pattern is consistent in the other comparisons (Supplementary Fig. S6), demonstrating that QAFI predictions, in addition to offering a numerical view of variant impacts, reach an accuracy level that allows it to contribute valuable insights to the variant classification problem.

## Discussion

This study introduces QAFI, a novel methodology (Fig. 1B) designed to improve the quantitative prediction of the functional impact of missense variants, an important goal in the evolution of in silico pathogenicity predictors (Masica and Karchin 2016; Diaz et al. 2023) that clears the way for novel applications. For example, continuous scores can serve to predict disease severity in some diseases, like in the case of ARSA (Trinidad et al. 2023) where it has fueled the corresponding CAGI6 challenge. They can also contribute to build more realistic fitness models in evolutionary studies, a field where in many cases only free energy estimates are employed (Echave and Wilke 2017).

At the core of our approach is the development and use of protein-specific predictors for the effects of variants. More precisely, we initially developed a set of thirty protein-specific predictors using multiple linear regression models that incorporate sequence and structure-based features. Some of these features were chosen based on our previous experience. For example, the Blosum62 substitution matrix, Shannon entropy, and PSSM, which we used to build the quantitative predictor for the BRCA1/2 proteins (Padilla et al. 2019) presented successfully at the CAGI5 ENIGMA challenge (Jain et al. 2024a). Shannon’s entropy and PSSM are two straightforward and intuitive ways to extract information from MSAs. However, it is important to note that other options are also viable for prediction purposes, such as the parameter used in the EA method, which is based on a sophisticated theoretical framework and has achieved notable results (Katsonis and Lichtarge 2017). To extend our initial set, we developed some additional features that capture the well-established principles (Kucukkal et al. 2015; Gerasimavicius et al. 2020; Cheng et al. 2021; Özkan et al. 2021), according to which the impact of variants is mainly due to destabilization effects or disruption of binding interactions. To this end, we chose properties that take into account the interactions of the native locus with its environment, an important component of protein stability (Serrano et al. 1992). This decision is illustrated by the use of the mean-force potential of Miyazawa and Jernigan (Miyazawa and Jernigan 1996). This coarse-grained statistical potential, based on a physicochemical formalism, gives us an approximate measure of the interactions of the native amino acid with its environment and is particularly easy to calculate. The step-wise behavior of this potential reduces the need to model the mutant structure, since its structural deviations from the native are unlikely to significantly change the pattern of interactions at the amino acid locus. Overall, the MJ potential has allowed us a more fruitful use of AlphaFold structures, going beyond the solvent accessibility calculations typically used as descriptors of variant impact, e.g., in Envision (Gray et al. 2018).

To train the protein-specific models we used available DMS data (Fig. 1A), and carefully cross-validated their performances, eliminating position-depending effects. The resulting models have better Pearson correlation coefficients than other methods, in 6 out of 14 proteins (Fig. 5A). In fact, in this benchmark, and together with AlphaMissense, protein-specific predictors are the technique with more per gene top-ranking performances; between both tools they essentially cover the full set of genes. However, it must be noted that in some cases the differences between these two, and between them and the remaining techniques, are minor (e.g., for TPMT, ADRB2, and SUMO1, Fig. 5A). In general, these results confirm the value of our set of features to model the impact of variants on protein function. However, the need for experimental datasets to create new protein-specific predictors restricts the extension of this approach to other proteins, a problem inherent to supervised methods (Diaz et al. 2023).

To address this generalization challenge we have changed our view on the protein-specific predictors, putting the focus on their ability to capture the common biophysics and biochemical principles underlying the impact of missense variants, rather than in their ability to reflect the concrete characteristics of a training protein. This perspective, inspired by the cross-prediction analyses (Fig. 3), has allowed us to make a conceptual leap from treating protein-specific models as solutions for distinct systems to viewing them as approximate solutions to the general prediction problem. This shift paves the way to apply ensemble learning principles to combine the different predictors (Bishop 2006), selecting the best ten for our ensemble model.

The results obtained (Figs. 2, 4 and 5) support the validity of our approach, by demonstrating QAFI’s competitive predictive capabilities for the tested proteins, even after an understandable, modest drop relative to the protein-specific tools and other binary classifiers. Specifically, this is the overall view in terms of the Pearson correlation. The behavior measured by the MAE values shows that although the accuracy of QAFI’s impact estimates has also decreased, QAFI results are essentially higher, or on par, with those of the remaining methods. Only Envision, specifically trained for quantitative predictions has a behavior similar to that of QAFI. The results for MAE are important because the specific reproduction of observed values is our goal when building quantitative predictors and plays an important role in their applications. An additional aspect that is worth noting, is that we compare the performance of QAFI with that of other tools (Fig. 5), a similar pattern emerges: (i) there is consistency among methods regarding the performance across different proteins, meaning that variants in some proteins are easier to predict than others; and (ii) although our models often outperforms other methods, this is not uniformly the case for all proteins. In fact, the prevailing pattern is that these tools outperform each other depending on the specific protein.

Building on the foundation of our generalization strategy, we tested QAFI during the CAGI6th edition. For the ARSA challenge, we used a set of pathogenic and benign variants for that protein that were previously available, allowing us to compare the potential performance of different QAFI versions (Fig. 6A). The tested versions included combinations of QAFI with some pre-existing tools (EVE, Envision, and REVEL), as well as QAFI models using a varying number of protein-specific predictors (10 and 30). This rigorous comparative analysis led to a ranking where the combination of QAFI + EVE + Envision + REVEL was manually selected as the top choice. As we await the final evaluation results by the CAGI6 assessors, it is noteworthy that the QAFI versions show Pearson correlations with the experimental results. (Trinidad et al. 2023) that are within the high range observed for our method (Supplementary Table S5).

To further validate our methodology, QAFI was applied to a large dataset of clinically annotated variants (Pejaver et al. 2022). By discretizing its scores and comparing them against known clinical annotations, we confirmed QAFI’s predictive accuracy and gained insights into its potential clinical applications. In particular, we observed that although QAFI’s overall performance ranked third when compared to a set of representative methods, it demonstrated improvements in correctly classifying variants that were misclassified by the top-ranking tools (Fig. 7C-D, Supplementary Fig. S6). Interestingly, a substantial number of these cases occurred in the rejection zones of these methods, as defined in (Pejaver et al. 2022) (Fig. 7D) thus indicating that QAFI can contribute to alleviate the problem of variants of uncertain significance (VUS).

A promising aspect of QAFI’s performance is its potential to generate predictions of gain-of-function (GOF) variants with increased activity (hypermorphic mutations (Backwell and Marsh 2022). This capability is not only valuable in clinical genetics but also crucial for protein engineering studies (Gelman et al. 2021; Luo et al. 2021). Unfortunately, identifying them is a weak spot in computational pathogenicity prediction (Reeb et al. 2020; Gerasimavicius et al. 2022). Unlike many computational pathogenicity predictors that assign equal scores to loss-of-function and gain-of-function variants, QAFI naturally produces values above 1 for hypermorphic cases, like Envision, facilitating their identification.

### Implications for future research

While QAFI represents a significant step forward, the variability in its performance across different proteins highlights areas for future development. Enhancing its model, or devising alternative models for different protein types, to consistently cover more proteins without losing accuracy will be a primary focus. Additionally, integrating QAFI with other available tools is an important goal for us, that could broaden its applicability, to find a more comprehensive computational solution to the variant interpretation problem.

## Electronic supplementary material

Below is the link to the electronic supplementary material.

Supplementary Material 1

Supplementary Material 2

Supplementary Material 3

Supplementary Material 4

Supplementary Material 5

Supplementary Material 6

## Data Availability

The datasets generated during and/or analysed during the current study are available through the Supplementary Tables. All the code used in this study to obtain the chosen features, train the protein-specific predictors and QAFI model, reproduce the feature importance analysis, together with an exhaustive in silico mutagenesis experiment (all possible amino acid substitutions at all residue locations across a set of 3,460 proteins, totaling 45,165,679 variants, and covering most of the Clinical Genome) is available at https://github.com/selenozkan/QAFI.
