# Supplementary material for: QAFI: a novel method for quantitative estimation of missense variant impact using protein-specific predictors and ensemble learning
Source: Hum Genet. 2024 Jul 24;144(2-3):191–208. doi: 10.1007/s00439-024-02692-z (PMC11976337; doi:10.1007/s00439-024-02692-z)
Supplement: Supplementary file 6 — Supplementary Material 6 [file 439_2024_2692_MOESM6_ESM.pdf]

SUPPLEMENTARY INFORMATION

QAFI: A Novel Method for Quantitative Estimation of Missense Variant Impact Using Protein-Specific Predictors and Ensemble Learning

Selen Ozkan[1], Natàlia Padilla[1], Xavier de la Cruz[1,2*]

[1]Research Unit in Clinical and Translational Bioinformatics, Vall d'Hebron Institute of Research (VHIR), Universitat Autònoma de Barcelona. Barcelona. Spain.

[2]Institució Catalana de Recerca i Estudis Avançats (ICREA), Barcelona, Spain.

*Correspondence: xavier.delacruz@vhir.org

Supplementary Figures

Fig. S1. Procedure for determining the ten most effective predictors for QAFI predictor.

Fig. S2. Median prediction error for the twenty natural native amino acids. View from the native amino acid perspective.

Fig. S3. Median prediction error for the twenty natural native amino acids. View from the mutant amino acid perspective.

Fig. S4. Performance analysis.

Fig. S5. Feature importance analysis.

Fig. S6. Distribution of correct predictions by QAFI relative to the score distributions of other pathogenicity predictors.

Supplementary Tables (the corresponding files are uploaded separately)

Table S1. Description of the thirty Deep Mutational Scanning (DMS) assays used in this study. Columns are: (1) UniProt ID, (2) Gene Symbol, (3) Protein Name, (4) Organism, (5) Total number of missense variants, (6) Origin of the data, (7) Reference.

Table S2. Experimental and predicted values for the whole variant dataset of the thirty proteins used to develop the protein-specific predictors. Columns are: (1) UniProt ID, (2) Gene Symbol, (3) Variant description, (4) Normalized value of the variant's experimental score, (5) Auto-prediction value (result of applying the protein-specific predictor of the protein in a cross-validation setting; see article's text), (6) QAFI prediction, (7-20) Fourteen features used in our models.

Table S3. Performance of the thirty protein-specific predictors. Columns are: (1) UniProt ID, (2) Gene Symbol, (3) Pearson correlation, (4) Spearman Rank correlation.

Table S4. Performance of QAFI and the tools shown in Fig 5. Performance of QAFI and the tools shown in Fig 5. Column 1: UniProt ID, (2) Gene Symbol. Columns 3-8 show the Pearson correlations for the following predictors: Protein-specific predictors (PSP), QAFI, Envision, EVE, AlphaMissense, ESM1b. Columns 9-14 show the Mean Absolute Errors for the same predictors.

Table S5. Performance of the six QAFI versions submitted to the CAGI6 ARSA challenge, sorted according to a visual prioritization process based on Fig. 6A. The Pearson and Spearman correlations were obtained after the experimental results were published. Columns are: (1) Model number and predictors contributing to the model, (2) Pearson correlation, (3) Spearman Rank correlation.

SUPPLEMENTARY FIGURES

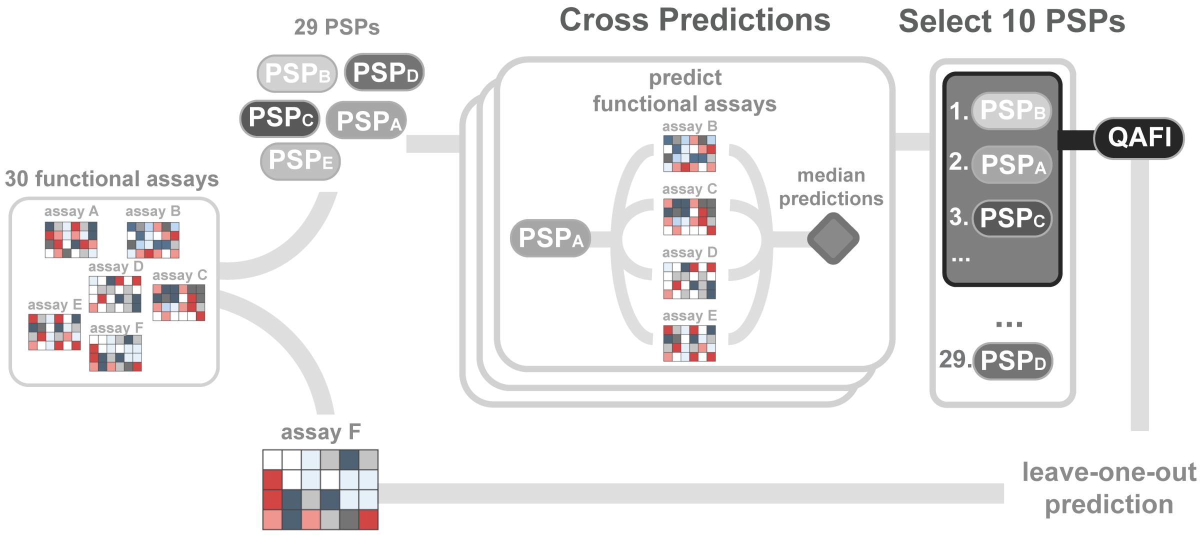

Fig. S1. Procedure for determining the ten most effective predictors for the QAFI predictor. In the figure, we illustrate the selection process used, which involved a specialized leave-one-out cross-validation procedure. (Note: each protein is represented by its corresponding DMS experiment). In each validation round, one protein was omitted from the dataset, and the performance of the protein-specific predictors (PSP) on the remaining twenty-nine proteins (DMS experiments) was assessed. These predictors were ranked based on their median Pearson correlation across the proteins within the training set, excluding their respective training protein. The performance of each predictor was recorded over the twenty-nine rounds in which it was part of the training set. The ten predictors most consistently demonstrating the highest performance were selected for the final version of QAFI. Note that in each round, the performance of the QAFI version applied to the left-out protein, was used to produce the results shown in Fig. 4.

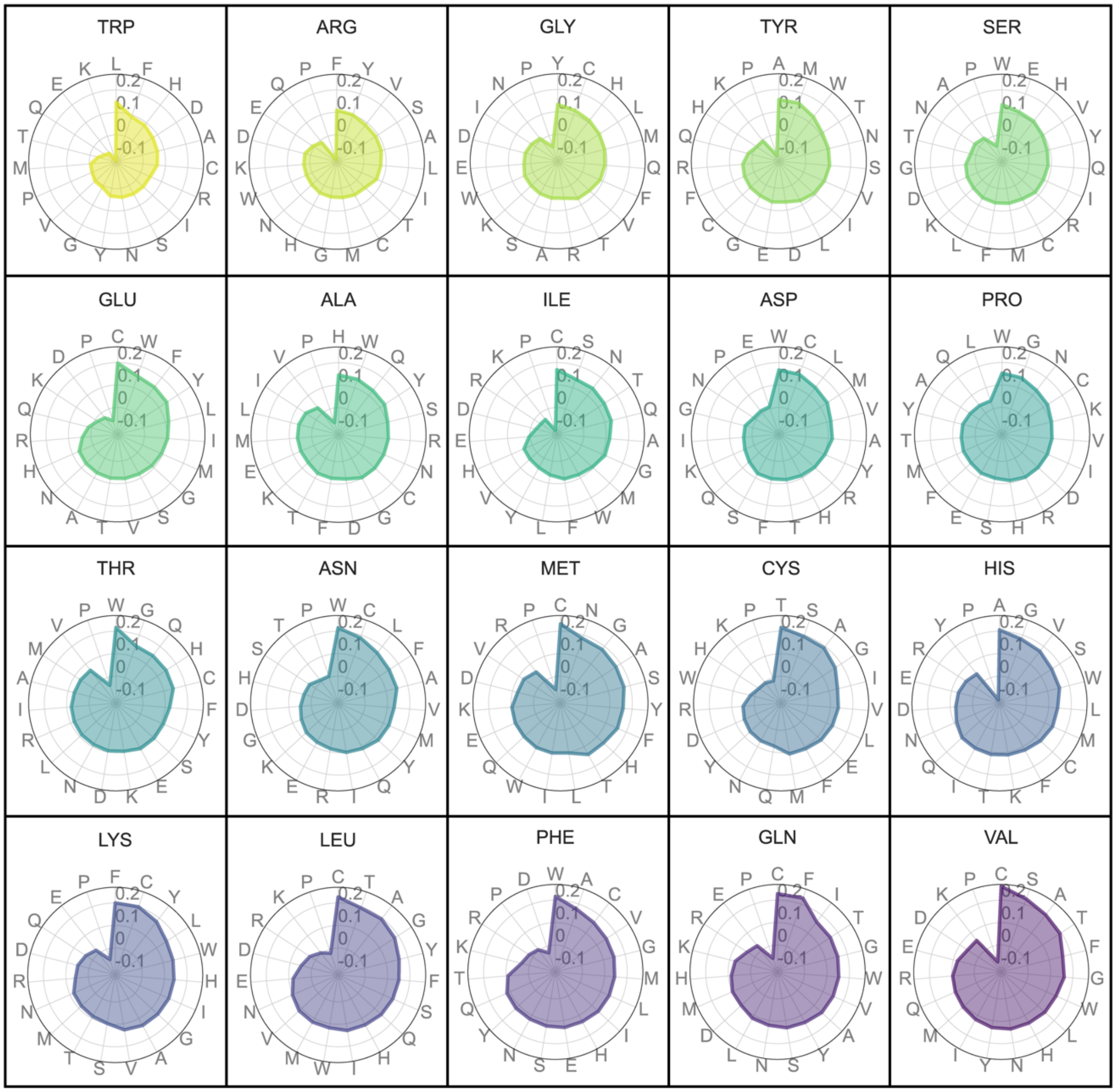

Fig. S2. Median prediction error for the twenty natural native amino acids. View from the native amino acid perspective. Each radar plot corresponds to one of the twenty natural amino acids, identified by its three-letter code. The plots are organized by increasing overall median error values for each amino acid, with colors indicating this ranking. A condensed version of this plot is presented in Fig. 2B.

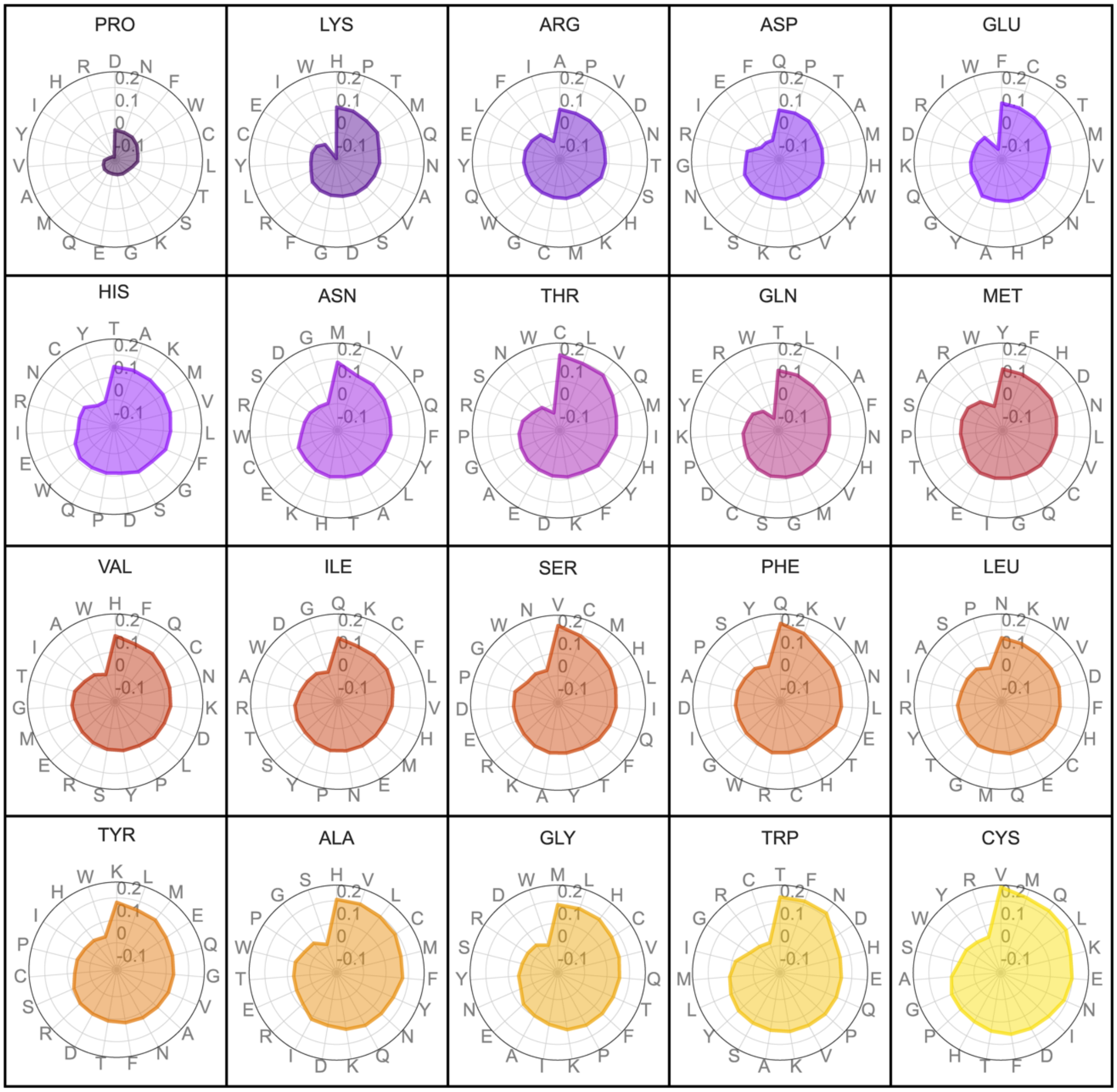

Fig. S3. Median prediction error for the twenty natural native amino acids. View from the mutant amino acid perspective. Each radar plot corresponds to one of the twenty natural amino acids, identified by its three-letter code. The plots are organized by increasing overall median error values for each amino acid, with colors indicating this ranking. A condensed version of this plot appears in Fig. 2C.

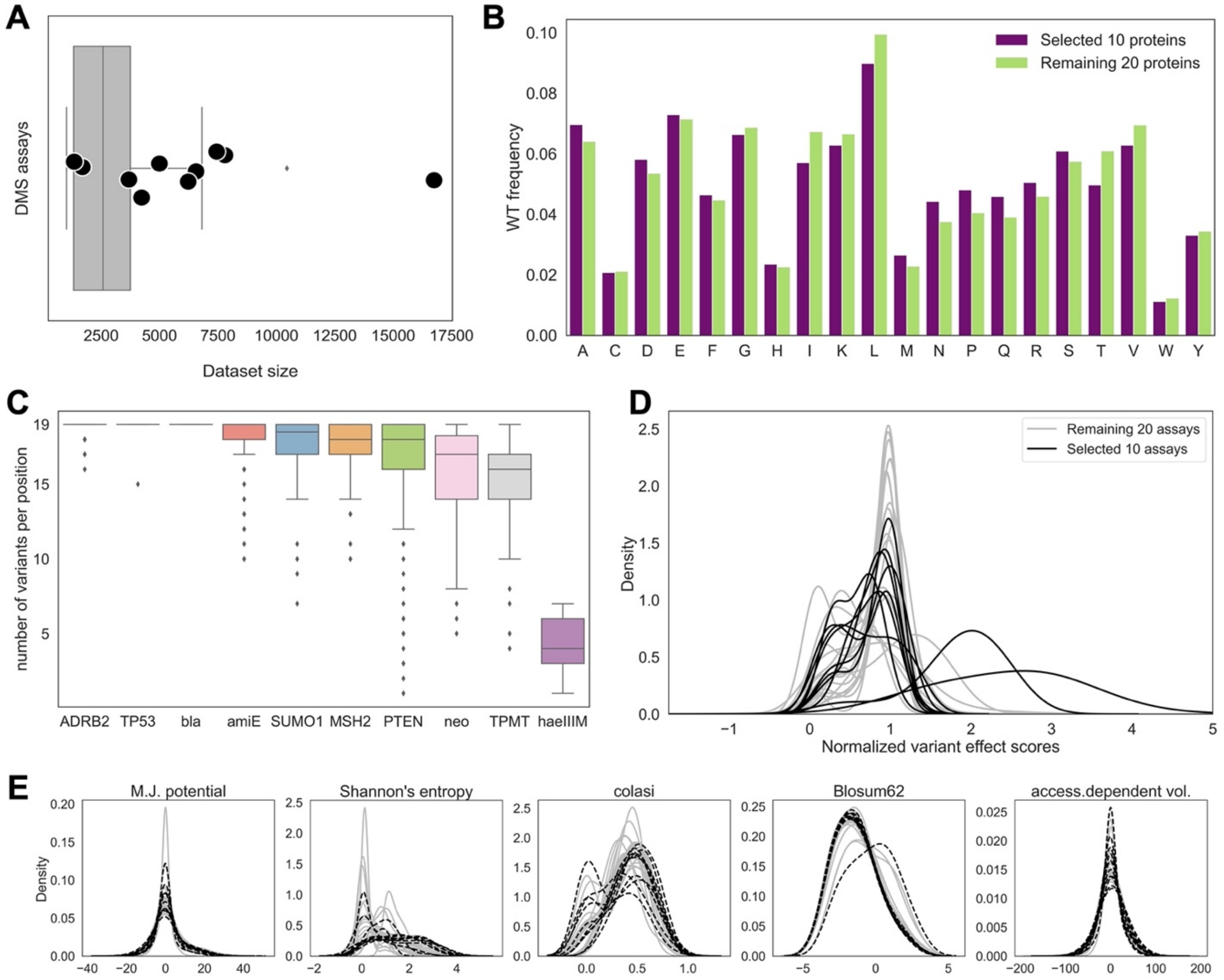

Fig. S4. Performance analysis. The figures cover five different aspects of the analysis conducted to identify the main factors contributing to QAFI's performance. (A) Number of variants in the DMS assays for the ten proteins at the core of QAFI (black circles) and the twenty remaining proteins (grey boxplot) in our protein dataset. (B) Wild-type amino acid composition of the ten (magenta) and twenty (green) proteins. (C) Number of variants in the DMS assays for the ten proteins; each boxplot corresponds to one of them. (D) Distribution of the normalized functional scores of the DMS assays for the ten (black) and twenty (grey) proteins. (E) Value distribution for five highly discriminant features for the ten (black) and twenty (grey) proteins.

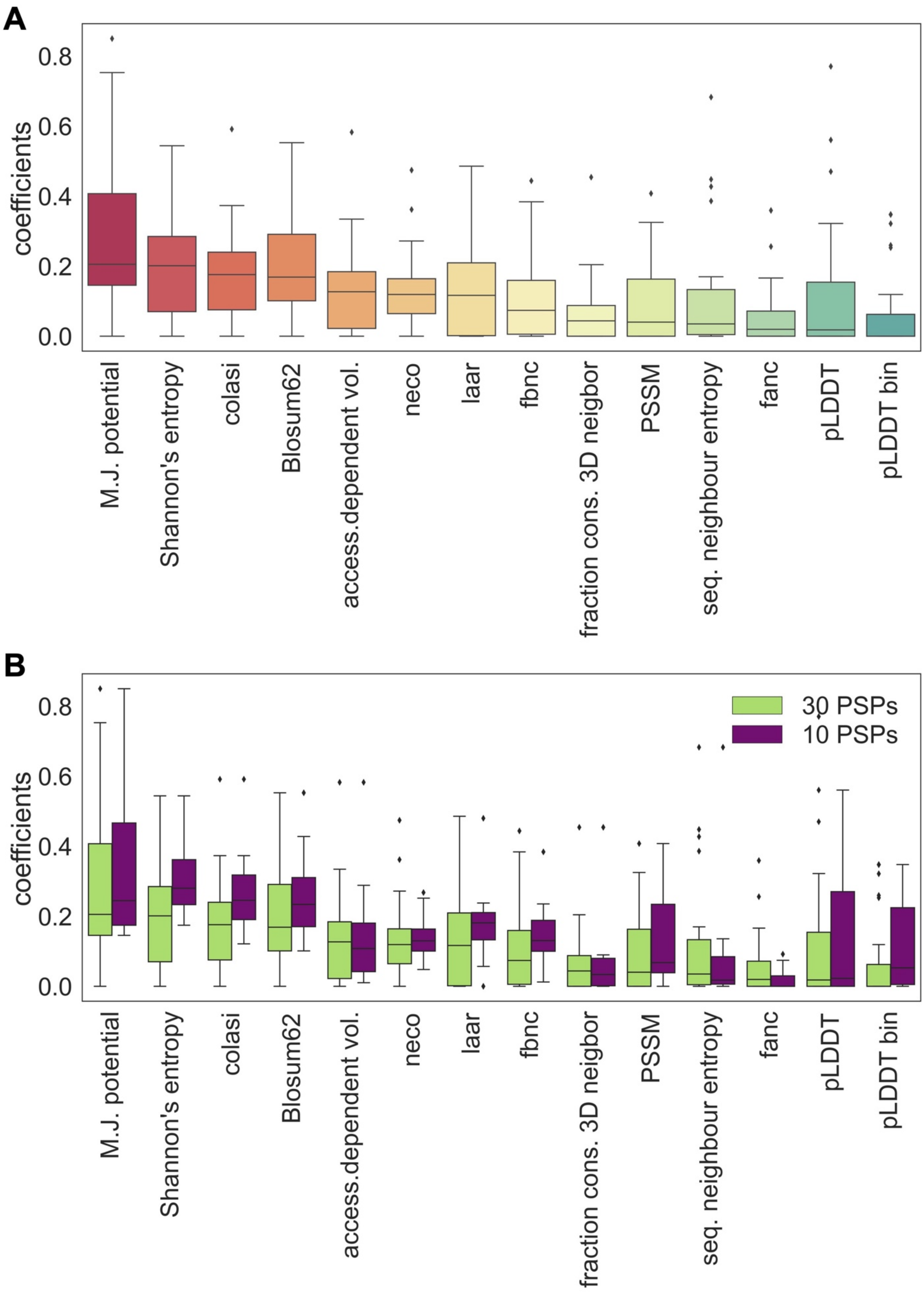
A
B
coefficients
0.0
0.2
0.4
0.6
0.8
30 PSPs
10 PSPs
M.J. potential
Shannon's entropy
colasi
Blosum62
access.dependent vol.
neco
laar
fbnc
fraction cons. 3D neigbor
PSSM
seq. neighbour entropy
fanc
pLDDT
pLDDT bin

Fig. S5. Feature importance analysis. (A) Distribution of the feature weights in the thirty protein-specific models for the proteins in our dataset. Each boxplot shows the distribution for a specific feature. The names of the features are on the horizontal axis. (B) Similar analysis as in (A), but here we show data for two protein populations. In green, we show again the boxplots for the thirty proteins, and in magenta, we show those corresponding to the subset of ten proteins forming the core of QAFI.

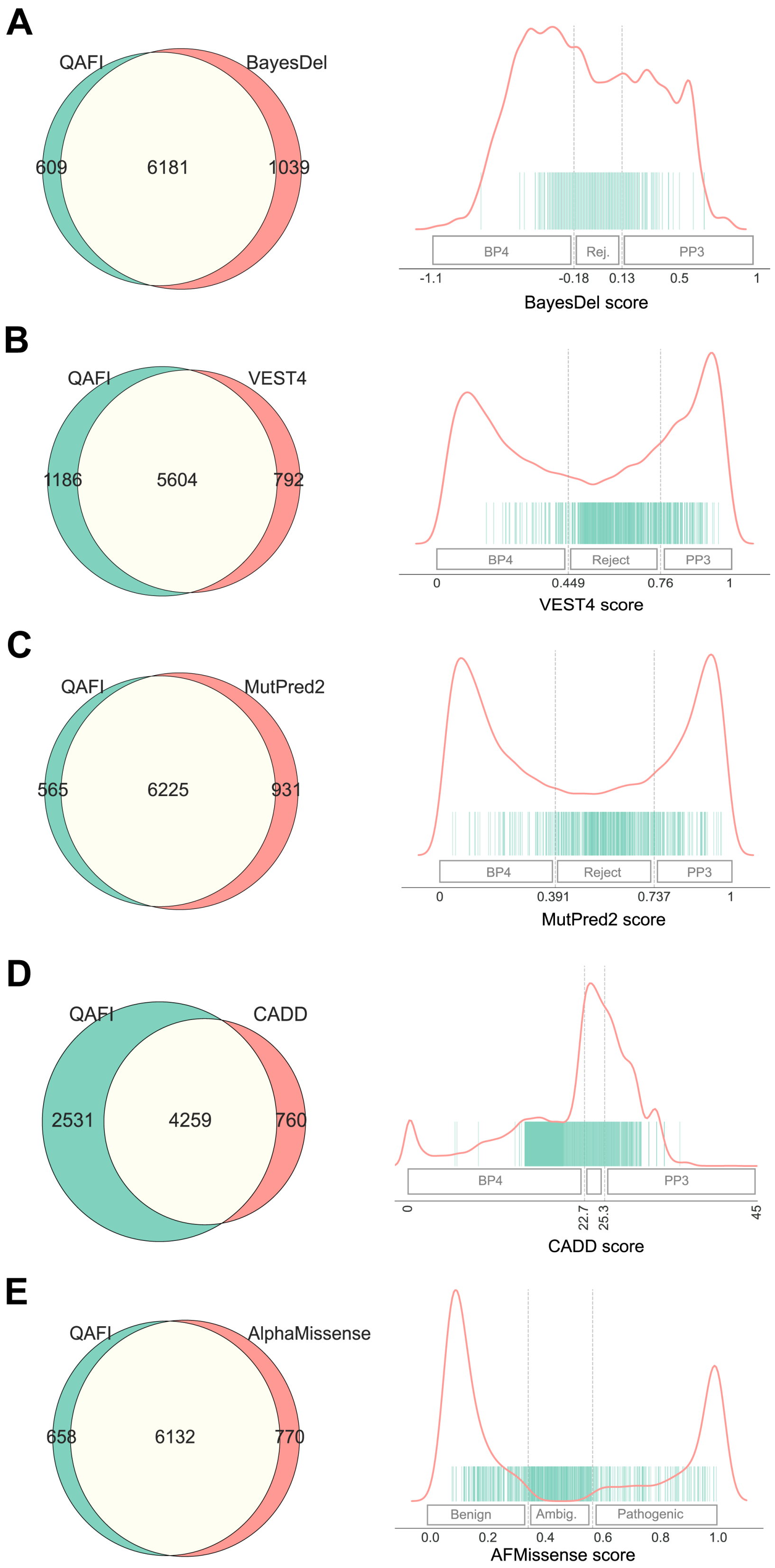
A
QAFI
BayesDel
609
6181
1039
BP4
Rej.
PP3
-1.1
-0.18
0.13
0.5
1
BayesDel score
B
QAFI
VEST4
1186
5604
792
BP4
Reject
PP3
0
0.449
0.76
1
VEST4 score
C
QAFI
MutPred2
565
6225
931
BP4
Reject
PP3
0
0.391
0.737
1
MutPred2 score
D
QAFI
CADD
2531
4259
760
BP4
PP3
0
22.7
25.3
45
CADD score
E
QAFI
AlphaMissense
658
6132
770
Benign
Ambig.
Pathogenic
0.0
0.2
0.4
0.6
0.8
1.0
AFMissense score

Fig. S6. Distribution of Correct Predictions by QAFI Relative to Other Pathogenicity Predictors. This figure compares QAFI's performance with that of BayesDel (A), VEST4 (B), MutPred2 (C), CADD (D), and AlphaMissense (E). Each panel consists of two parts: on the left, a Venn diagram illustrates the complementarity between QAFI and the respective predictor. Yellow indicates variants correctly predicted by both tools; green and orange denote variants uniquely predicted by QAFI and the other method, respectively. On the right, the distribution of QAFI's correct predictions (green vertical lines) is shown relative to the score distribution of the other predictor (orange curve). For panels A-D, boxes above the axis delineate evidence regions for the predictor's scores used in clinical annotation of variants, as defined by Pejaver et al. (2023). For panel E, the boxes represent regions defined by AlphaMissense, according to Cheng et al. (2023).

REFERENCES

Cheng J, Novati G, Pan J, et al (2023) Accurate proteome-wide missense variant effect prediction with AlphaMissense. Science 381:eadg7492. https://doi.org/10.1126/science.adg7492

Pejaver V, Byrne AB, Feng B, et al (2022) Calibration of computational tools for missense variant pathogenicity classification and ClinGen recommendations for PP3 / BP4 criteria. Am J Hum Genet 109:2163–2177. https://doi.org/10.1016/j.ajhg.2022.10.013
